# Human Herpesvirus 6 and Malignancy: A Review

**DOI:** 10.3389/fonc.2018.00512

**Published:** 2018-11-13

**Authors:** Eva Eliassen, Emily Lum, Joshua Pritchett, Joseph Ongradi, Gerhard Krueger, John R. Crawford, Tuan L. Phan, Dharam Ablashi, Stanley David Hudnall

**Affiliations:** ^1^HHV-6 Foundation, Santa Barbara, CA, United States; ^2^Department of Internal Medicine, Mayo Clinic, Rochester, MN, United States; ^3^Institute of Medical Microbiology, Semmelweis University, Budapest, Hungary; ^4^Department of Pathology and Laboratory Medicine, University of Texas- Houston Medical School, Houston, TX, United States; ^5^Department of Neurosciences and Pediatrics, University of California San Diego and Rady Children's Hospital, San Diego, CA, United States; ^6^Department of Microbiology and Immunology, Tulane University School of Medicine, New Orleans, LA, United States; ^7^Department of Pathology, Yale University, New Haven, CT, United States

**Keywords:** HHV-6, herpesvirus, human herpesvirus 6, HHV6, oncogenic, cancer, malignant, transformation

## Abstract

In order to determine the role of human herpesvirus 6 (HHV-6) in human disease, several confounding factors, including methods of detection, types of controls, and the ubiquitous nature of the virus, must be considered. This is particularly problematic in the case of cancer, in which rates of detection vary greatly among studies. To determine what part, if any, HHV-6 plays in oncogenesis, a review of the literature was performed. There is evidence that HHV-6 is present in certain types of cancer; however, detection of the virus within tumor cells is insufficient for assigning a direct role of HHV-6 in tumorigenesis. Findings supportive of a causal role for a virus in cancer include presence of the virus in a large proportion of cases, presence of the virus in most tumor cells, and virus-induced *in-vitro* cell transformation. HHV-6, if not directly oncogenic, may act as a contributory factor that indirectly enhances tumor cell growth, in some cases by cooperation with other viruses. Another possibility is that HHV-6 may merely be an opportunistic virus that thrives in the immunodeficient tumor microenvironment. Although many studies have been carried out, it is still premature to definitively implicate HHV-6 in several human cancers. In some instances, evidence suggests that HHV-6 may cooperate with other viruses, including EBV, HPV, and HHV-8, in the development of cancer, and HHV-6 may have a role in such conditions as nodular sclerosis Hodgkin lymphoma, gastrointestinal cancer, glial tumors, and oral cancers. However, further studies will be required to determine the exact contributions of HHV-6 to tumorigenesis.

## Background

Human herpesvirus-6A and−6B (HHV-6A and HHV-6B) are linear, double-stranded DNA viruses and members of betaherpesvirinae, along with CMV and HHV-7. HHV-6A and HHV-6B were identified as two distinct herpesviruses as early as 1992 (1), and in 2014, they were formally classified as two separate species (2). While less is known about the epidemiology of HHV-6A, HHV-6B is a ubiquitous virus, with over 90% of the human population infected within the first 3 years of life. In 1986, HHV-6 was isolated from the peripheral blood mononuclear cells of AIDS-associated non-Hodgkin lymphomas by Robert Gallo and associates at the National Cancer Institute in their search for undiscovered herpesviruses that might be causing cancer in HIV-infected patients (3). Soon afterward, it was found that both HHV-6-transfected NIH3T3 fibroblasts (4) and human primary foreskin epidermal keratinocytes transfected with HHV-6 subgenomic clones PZV714 (5) and PZVB70 (6) produced tumors when injected into nude mice (7). The PZV714 and PZVB70 tumors were cytogenetically abnormal, with loss of chromosomes 12 and 13 and acquisition of extra marker chromosomes. These early results suggested that HHV-6 may be oncogenic in some settings. Decades later, several studies identified HHV-6A and HHV-6B (2) within a wide variety of tumors, including glioma, oral cancer, cervical cancer, adrenocortical tumors, gastrointestinal cancer, classical Hodgkin lymphoma, and non-Hodgkin lymphoma, as summarized in Tables 1–**8** (99).

**Table 1:** Detection of HHV-6 in tumor tissue from patients with Hodgkin Lymphoma.

**References**	**Detection method**	**HHV-6 positive patients/Total number of patients (%)**	**HHV-6 positive controls/Total number of controls (%)**	**Type of control tissue**	**Of typed samples: HHV-6A%, HHV-6B%, (HHV-6A/B Coinfection%); Coinfections with other viruses**	**% Of positive samples belonging to the nodular sclerosis subtype**	**Notes**
Kiani et al. (8)	PCR	12*/22 **(54%)**	NA	NA	83%, 0%, (17%)	42%	*5/9 NS, 5/11 MC, and 2/2 LP
Siddon et al. (9)	PCR	27/31 **(87%)**	5/6 (83%)	RH	40%, 30%, (30%)	100%	NA
Lacroix et al. (10)	PCR	68/86 **(79.1%)**	NA	NA	7%, 93%	83.6%	NA
Hernández-Losa et al. (11)	PCR	8/20 **(40%)**	17*/52 (33%)	Donor spleen lymphocytes, reactive lymphadenitis	88% EBV+	NA	NA
Collot et al. (12)	PCR	13/37 **(35.1%)**	51/68 (75%)	Normal saliva	8%, 92%	100%	NA
Shiramizu et al. (13)	PCR	0/47 **(0%)**	NA	NA	NA	NA	NA
Schmidt et al. (14)	PCR	11*/88 **(13%)**	NA	NA	73%, 27%; 64% HHV-7+, 64% EBV+, 22% CMV+	45%	*5 NS, 4 MC, 2 untyped
Valente et al. (15)	PCR, SB	38*/52 **(73%)**	13/19 (68.4%)	Reactive LNs	5%, 95%	79%	*73.1% of NS cases, 50% of interfollicular, 70% of MC, and the single case of LD
Carbone et al. (16)	PCR	0/5 **(0%)**	NA	NA	NA	NA	NA
Razzaque et al. (17)	PCR	1/1 **(100%)**	0/3 (0%)	Normal LNs	NA	NA	NA
Dolcetti et al. (18)	PCR	3/10 **(30%)** HIV+, 13/43 **(30%)** HIV-	15*/30 (50%)	Reactive LNs	HIV+: 0%, 67%, (33%), HIV-: 8%, 92%	NA	*12 HHV-6B, 1 A/B coinfection in HIV+ controls; 2 HHV-6B in HIV- controls
Di Luca et al. (19)	PCR	13/45 **(29%)**	8/45 (17%)	Healthy donor PBLs	8%, 92%	NA	NA
Trovato et al. (20)	PCR, ISH	1/15 **(6.7%)**	NA	NA	EBV+	NA	NA
Sumiyoshi et al. (21)	PCR, SB	9/14 **(64.3%)**	55/56 (98.2%)	Reactive lymphadenopathy	NA	NA	NA
Gledhill et al. (22)	SB	0/35 **(0%)**	NA	NA	NA	NA	NA
Torelli et al. (23)	PCR, SB	3/25 **(12%)**	NA	NA	NA	100%	NA
Jarrett et al. (24)	PCR	0/29 **(0%)**	0/35 (0%)	Nonlymphomatous tissues*	NA	NA	*Reactive LNs, cancers, neoplasms, sarcoidosis, Sjögren's syndrome, and miscellaneous
Siddon et al. (9)	IHC^a^	18/21 **(86%)**	NA	NA	50%, 33%, (17%)	100%	Antigen found in RS cells in 48% NSHL cases. Scattered positive RS cells also positive for p41, p98, U94. By FISH, normal tonsil showed rare scattered HHV-6, whereas HL showed numerous scattered cells harboring multiple copies of HHV-6
Lacroix et al. (25)	IHC^b^, RS cells^1^	28/38 **(73.7%)**	NA	NA	0%, 100%	NA	In 17/28 patients (60.7%), DR7B was detected only in RS cells. Mummified cells were DR7B+ in 9/28 cases (32.1%). DR7B detected in 9/9 HHV-6+/EBV+ NSHL patients
Lacroix et al. (25)	IHC^c^, RS cells^1^	15/38 **(39.5%)**	NA	NA	NA	NA	NA
Lacroix et al. (25)	IHC^c^, infiltrating cells^1^	38/38 **(100%)**	NA	NA	NA	NA	NA
Luppi et al. (26)	IHC^d^, RS cells^1^	2/14 **(14.3%)**	0*/16 (0%)	Non-neoplastic LNs^1^	NA	100%	Mummified RS cells were positive. Only rare cells (reactive histiocytes, plasma cells) had cytoplasmic reaction for 101K, gp116. *In most controls, only isolated cells stained for p101K, gp106, and gp116
Luppi et al. (26)	IHC^e^, RS cells^1^	0/14*(0%)	NA	NA	NA	NA	Rare cells (reactive histiocytes, plasma cells) had cytoplasmic reaction for 101K, gp116
Valente et al. (15)	ISH	47/57 **(82.4%)**	NA	NA	NA	70%	No specific localization in neoplastic tissue. No HD or RS cells were positive
Razzaque et al. (17)	IHC	0/1 **(0%)**	NA	NA	NA	NA	Monocytic cells including HD and RS cells most frequently positive, less frequently lymphoid cells
Krueger et al. (27)	IHC^d^	**(37%)**	NA	NA	NA	NA	Monocytic cells including HD and RS cells most frequently positive, less frequently lymphoid cells
Krueger et al. (27)	IHC^c^	**(63%)**	NA	NA	NA	NA	NA
Rojo et al. (28)	ISH	14/15 **(93.3%)**	13/13 (100%)	LNs from APL and RH	NA	21%	Lymphoid cells, monocytes most often positive, RS cells sometimes positive. Monocytes expressed antigens. Rarely, RS cells also expressed antigens (most markedly HAR3, less frequently p41). 14/15 cases had increased numbers of HHV-6 DNA containing cells with values between 22 and 590 times over those in RH (median 188)

*APL, atypical polyclonal lymphoproliferation; EBV, Epstein-Barr virus; FISH, fluorescence in situ hybridization; HL/HD, Hodgkin lymphoma/Hodgkin disease; IHC, immunohistochemistry; ISH, in situ hybridization; LD, lymphocyte depleted; LN, lymph node; LP, lymphocytic predominance; MC, mixed cellularity; NA, not available; NS, nodular sclerosis; PBLs, peripheral blood lymphocytes; RH, reactive lymphoid hyperplasia; RS, Reed Sternberg; SB, Southern blot*.

^*^For those studies that employed both PCR and Southern Blot or ISH, only PCR results are presented.

^*^If quantitative data is unavailable for any given method or for controls, the data is excluded.

^*^Cao et al. (29) and Cantalupo et al. (30) both analyzed samples from the same database containing deep-sequencing reads from human tumors.

^*^IHC was performed using antibodies against

a *whole virus lysate*,

b *DR7B*,

c *gp116/64/54*,

d p41, and

e *gp106*.

1 *Samples HHV-6 positive by PCR*.

*Bold values represent the percentage of HHV-6-positive samples among samples tested*.

Both HHV-6A and HHV-6B share similar replication cycles: immediate-early (IE) proteins are synthesized within a few hours post-infection, which regulate the expression of the early and late genes. It takes ~72 h to complete a replication cycle (i.e., from infection to new virion release). It is now known, however, that these two species utilize distinct receptors for cellular entry: HHV-6A uses CD46, a ubiquitous complement regulatory protein, whereas HHV-6B primarily uses CD134, a molecule expressed only on activated T cells (100). Like other herpesviruses, HHV-6 displays broad cellular tropism, although it replicates most efficiently in CD4^+^ T cells *in vitro*. As is the case for other oncogenic human herpesviruses, including Epstein-Barr virus (EBV) and HHV-8, also known as Kaposi's sarcoma-associated herpesvirus (KSHV), HHV-6 establishes latency in lymphocytes and possesses a strong immunomodulatory capacity that can trigger both immunosuppressive and chronic inflammatory pathways (101).

HHV-6A/B are unique among human herpesviruses in their ability to integrate into the telomeres of chromosomes as a form of latency. They share significant homology with a neoplastic avian alpha herpesvirus, Marek's disease virus (MDV), which also integrates into the subtelomeric region of the chromosome and causes an aggressive T-cell lymphoma and immunosuppression in domestic chickens (102, 103). Approximately 1% of the world's population carries inherited chromosomally integrated HHV-6 (iciHHV-6), with the full genome integrated into the subtelomeric region of the chromosome in every nucleated cell (104). These integrated genomes can be activated by drugs (105), and immunocompromised patients with iciHHV-6 can develop symptomatic infections from the integrated strain (106). Inherited ciHHV-6 can affect telomeric stability (107), and telomeric disruption has been associated with hematologic diseases, such as aplastic anemia (108). Recent studies suggest that iciHHV-6 can be reactivated by HDAC inhibitors and can be horizontally transmitted through liver transplantation (109, 110).

Even if not directly oncogenic, HHV-6 may contribute to oncogenesis in cooperation with other viruses, such as EBV and human papillomavirus (HPV) (99). The molecular basis of this phenomenon is only partially understood. Herein, we review the wide range of neoplastic conditions associated with HHV-6 infection and the possible mechanisms by which HHV-6 might contribute to tumorigenesis.

## Methodological limitations

The early use of such methods as serological testing and qualitative PCR represented pioneering steps in elucidating the roles of HHV-6 in a range of diseases, including several types of cancer. Data resulting from these techniques was largely inconclusive, and unfortunately, subsequent studies employing more targeted approaches, such as IHC and ISH, have not been carried out as frequently as would be needed to gain a better understanding of HHV-6 in cancer. Consequently, the progress of research in this field has been slow. As a ubiquitous virus, HHV-6 can often be found at a low viral load in the blood, latent or slightly reactivated without deleterious effects. It also tends to reactivate during periods of immunosuppression and stress. Thus, it is difficult to distinguish background reactivation from pathological reactivation using serological and qualitative PCR approaches that do not provide information on viral load, viral transcription, viral species, and localization of the virus. In tissues, HHV-6 may be found in infiltrating lymphocytes; because of this, staining techniques are preferred over whole-tissue PCR, as infection of tumor cells can thereby be distinguished from infection of infiltrating cells, and active infection can be differentiated from latent virus.

The role of HHV-6 in other illnesses is still under investigation, which can be problematic when choosing controls. For example, HHV-6 has been investigated as a contributing/causative factor in cases of Alzheimer's disease, benign lymphoproliferative disorders, and certain autoimmune conditions. Until the relationships between HHV-6 and these conditions (as well as others) is better understood, it is unclear whether using samples from patients with these disorders as controls is appropriate. Likewise, using samples from immunocompromised individuals may inaccurately represent the HHV-6-status of healthy controls, as patients with compromised immunity are at a much greater risk of HHV-6 reactivation, which can lead to clinical manifestations in some instances. Whenever possible, matched normal tissues from healthy individuals should be used as controls in order to avoid overestimation of the prevalence and activity of HHV-6 in healthy persons.

Since early research into the ability of HHV-6 to induce transformation, little work has been done on this topic. The use of animal models could be especially useful in this area, but to date, few such models exist for the study of HHV-6. In going forward, it will be necessary to standardize reference materials (111), emphasize collaboration among laboratories, and perform *in vitro* experiments to characterize the transforming potential of HHV-6, including further analysis of the functions of the viral DR-7 gene, as well as any effects the pair of viruses may have on other oncogenic agents.

In light of these limitations, this review will focus on studies and case reports that have employed robust techniques that contribute to our understanding of the correlates of HHV-6 infection. In each section, we will not focus on studies relying on standard serological techniques, instead focusing on studies using PCR, immunohistochemistry (IHC), *in situ* hybridization (ISH), sequencing techniques, and other targeted approaches, and the greatest attention will be paid to those studies that differentiate between active and latent infection, allow localization of viral DNA/mRNA/proteins, and distinguish between HHV-6A and HHV-6B. Likewise, we will include studies of HHV-6 detection in hematological, neurological, gastrointestinal, gynecological, and head and neck cancers in a series of tables, while studies detailing the presence of HHV-6 in other cancers that are, at present, more weakly associated with the virus, are not included. Those cancers that do not fall under these five systems, including skin, lung, and urological cancers, have fewer than five studies published on their association with HHV-6 and are not included in the tables. The tables illustrate the great variation in HHV-6 detection across studies, highlighting the challenges involved in drawing conclusions from many cases, particularly from detection rates alone.

## HHV-6 as the sole infectious agent in cancers: by system/site

### Hematological

#### Hodgkin lymphoma (Table 1, Figure 1)

Using PCR, HHV-6 was detected in the lymph nodes of 13/31 (12 HHV-6B, 1 HHV-6A) nodular sclerosis cases but 0/6 tissues from mixed-cellularity and lymphocyte-depleted HL (12), and the mean copy number was high at 6,711.4 per μg of DNA. Other PCR-based studies also found HHV-6 in a proportion of cases, but those based on ISH/IHC have proven to be more fruitful. In 1994, Krueger et al. reported HHV-6 p41 and gp116/64/54 antigen in Hodgkin Reed-Sternberg (RS) cells (27). Four years later, Luppi et al. detected HHV-6 p41 early antigen in the so-called “mummified” RS cells in two Hodgkin disease cases (26). The presence of viral antigen in these HL-associated cells indicated possible viral contribution to HL.

**Figure 1:**
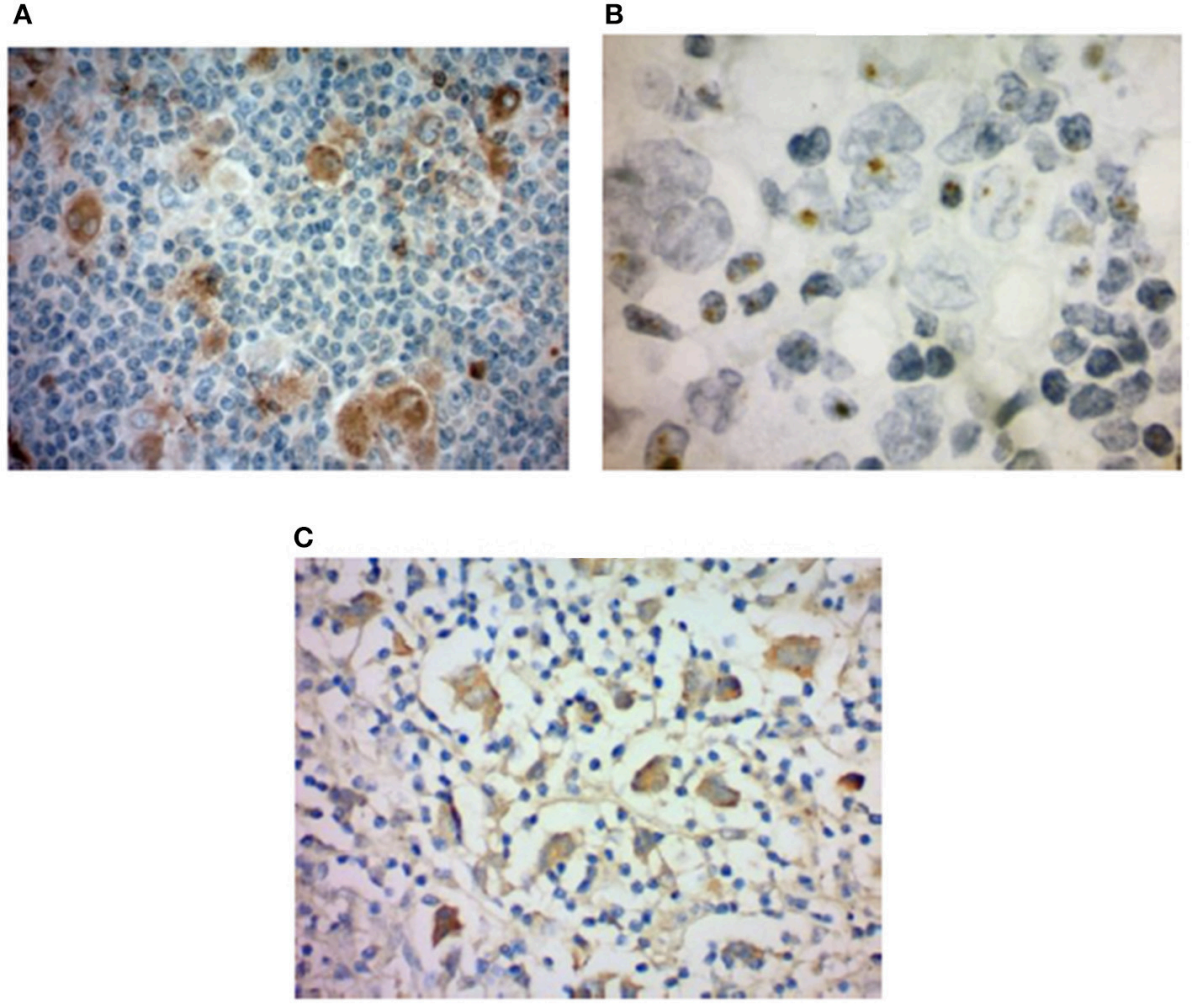
HHV-6 in nodular sclerosis Hodgkin lymphoma. **(A)** Presence of HHV-6 p41 by immunohistochemistry. Cytoplasmic staining of numerous large cells using a monoclonal antibody to HHV-6 p41 (3E3 clone). **(B)** HHV-6 U94 detection by immunohistochemistry. Staining with monoclonal antibody to HHV-6 U94 reveals positivity in the cytoplasm of numerous large cells. **(C)** Presence of HHV-6 by colorimetric *in-situ* hybridization with multiplex probe. HHV-6 DNA present in the nuclei of both small and large cells. From Eliassen et al. (112).

Later, Lacroix et al. analyzed lymph node biopsies from 48 patients, 38 of which were EBV-negative, and all but three of nodular sclerosis (NS) subtype (25). HHV-6 protein DR7B was detected by IHC in RS cells of 74% of EBV-negative cases, and in most of these cases (61%), DR7B was detected only in RS cells. Among the nine patients with HHV-6/EBV coinfections, the DR7B-positive RS cells were also positive for the EBV oncoprotein LMP-1, a key player in EBV-induced cell transformation. Notably, in 6 of the 9 patients with co-infections, DR7B was detected only in RS cells; all 6 patients had the NS subtype. In the single EBV/HHV-6+ patient with mixed cellularity subtype HL, only LMP-1 was found in RS cells, while DR7B was exclusively detected in infiltrating inflammatory cells. Both DR7A and DR7B have been shown to bind and inactivate the tumor suppressor p53 (113), suggesting that DR7 might play a role in lymphomagenesis (2).

The most recent study, conducted by Siddon et al., found HHV-6 by PCR in 27 of 31 (87%) NS subtype HL lymph node specimens. In nearly half of the HHV-6-positive cases (48%), U94 latent antigen, p41 early antigen, and/or p98 (HHV-6B) late antigen was expressed in scattered RS cells and leukocytes, suggesting viral replication within granulocytes and a few RS cells (9). Collectively, the use of immunohistochemical techniques has provided evidence suggestive of variable HHV-6 activity depending on HL subtype, with involvement of HHV-6 appearing more likely among NS cases. Additionally, greater viral load among NS cases than those of other subtypes supports the notion that there are subtype-specific differences in the role of HHV-6 in HL (10). Siddon et al. also reported that patients with HHV-6+ RS cells trended toward a younger age than those with EBV+ RS cells (*p* = 0.073).

The reported preferential localization of DR7 in RS cells, and not primarily among infiltrating inflammatory cells as would be expected as a normal distribution of latent virus or in the case of opportunistic reactivation, suggests that HHV-6 may be involved in HL pathogenesis (25). However, in some cases, HHV-6 was primarily located in infiltrating granulocytes, suggesting that HHV-6 may simply be reactivated within the immunodeficient microenvironment of HL. While greater sample sizes and further investigation is needed, the evidence suggestive of a role for HHV-6 in HL is presently strongest for NS cases.

#### Non-hodgkin lymphoma (Table 2)

In non-Hodgkin lymphoma (NHL), associations are weaker. The heterogeneity of methods, types of NHL, and types of controls included in past studies has compounded the difficulty in assigning a role to HHV-6 in this setting. PCR-based reports have documented an extreme range of HHV-6 positivity with 0–100% of blood/bone marrow/lymph nodes from cases testing positive. For instance, Usui et al. detected HHV-6 in up to 6.7% of ocular MALT tissues, while an earlier team documented a positive rate of 28.6% (31, 38). Among some studies, the ability to draw conclusions is limited by small sample sizes, even when the HHV-6 species is typed, the viral load is quantified, and types of lymphoma are differentiated (8, 12, 36). While the percentages of positive samples can be intriguing when viewed alone, the small sample sizes do not allow for adequate comparison. Lack of differentiation among NHL types in other studies also limits analysis.

**Table 2:** Detection of HHV-6 in tumor tissue from patients with Non-Hodgkin Lymphoma.

**References**	**Type of Lymphoma in patient cohort, if specified**	**Detection method**	**HHV-6 positive patients/Total number of patients (%)**	**HHV-6 positive controls/Total number of controls (%)**	**Type of control tissue**	**Of typed samples: HHV-6A%, HHV-6B%, (HHV-6A/B Coinfection%); Coinfections with other viruses**	**Notes**
Kiani et al. (8)	Not specified	PCR	8*/22 **(36%)**	NA	NA	100%, 0%	*6/10 cases diffuse large cell, 2/7 cases Burkitt lymphoma
Usui et al. (31)	Conjunctival MALT	PCR	1/19 **(5.2%)**	0/23 (0%)	Normal conjunctiva	NA	NA
Usui et al. (31)	Orbital MALT	PCR	1/15 **(6.7%)**	0/23 (0%)	Normal conjunctiva	NA	NA
Usui et al. (31)	Orbital DLBCL	PCR	0/7 **(0%)**	0/23 (0%)	Normal conjunctiva	NA	NA
Yoon et al. (32)	AITL	PCR	0/11 **(0%)**	4/66 (6.1%)	Lymphoma, MF, PL, and normal skin, LN tissues	NA	NA
Zhou et al. (33)	AITL	PCR	19/42 **(45.2%)**	NA	NA	0%, 100%; 89% EBV+	HHV-6 exclusively found in histological pattern II or III cases, and was present in significantly more cases with pattern III (17/29 = 58.6%) than pattern II (2/12 = 16.7%)
Kolonic et al. (34)	PMLBCL	PCR	1/24 **(4.2%)**	NA	NA	NA	NA
Hernández-Losa et al. (11)	B- and T-cell	PCR	14/63 **(22%)**	17/52 (33%)	Normal spleen lymphocytes, reactive lymphadenitis	Most HHV-6+ samples were EBV+	NA
Vrsalovic et al. (35)	AITL	PCR	4*/18 **(22%)**	NA	NA	25% EBV +	*TCR-gamma clonal
Collot et al. (12)	B-, T-, NK-cell	PCR	11*/49 **(22.4%)**	51/68 (75%)	Normal saliva	9%, 91%	*8 B-cell, 3 T-/NK-cell
Allory et al. (36)	HPS-associated B- and NK-cell	PCR	2*/3 **(66.7%)**	NA	NA	NA	*2/2 DLBCL
Asou et al. (37)	Various	PCR	28*/160 **(17.5%)**	2/31 (6.5%)	CD and reactive lymphadenopathy biopsies	PEL: HHV-8+ and EBV+ AIDS NHL: 25% EBV+	*0/10 (0%) MALTomas, 1/21 (5%) PELs, 19/104 (18%) non-AIDS NHL, 8/25 (32%) AIDS NHL
Daibata et al. (38)	Ocular MALT lymphoma	PCR	4/14 **(28.6%)**	NA	NA	25% EBV+	NA
Ohyashiki et al. (39)	T- and B-cell	PCR	5/14 **(35.7%)**	5/5 (100%)	IL and CD	0%, 100%	NHL viral load: 6.4–810 copies/microgram DNA Control viral load: 5.5–3,705 copies/microgram DNA
Dolcetti et al. (18)	HIV+, HIV-	PCR	1/17 **(6%)** HIV+, 0/35 **(0%)** HIV-	15*/30 (50%)	Reactive LNs	0%, 100%	*12 HHV-6B, 1 A/B coinfection in HIV+ controls; 2 HHV-6B in HIV- controls
Razzaque et al. (17)	T- and B-cell	PCR	6/6 **(100%)**	0/3 (0%)	Normal LNs	NA	NA
Valente et al. (15)	HIV+	PCR	10/15 **(66.6%)**	13/19 (68.4%)	Reactive LNs	NA	NA
Carbone et al. (16)	HIV+ SNCC, ALC	PCR	1*/14 **(7.1%)**	NA	NA	0%, 100%; EBV+	*SNCC
Di Luca et al. (19)	HIV-, HIV+^*^	PCR	1/45 **(2.2%)**	8/45 (17%)	Healthy donor PBLs	0%, 100%; HIV+	*35 HIV-, 10 HIV+
Brice et al. (40)	Cutaneous T-cell	PCR	1/30 **(3.3%)**	1/19 (5.3%)	LP and PL tissues	NA	NA
Luppi et al. (41)	AITL	PCR	7/12 **(58.3%)**	1/11 (9.1%)	Hyperplastic, non-hyperplastic LNs	40%, 40%, (20%); 57% EBV+	In one HHV-6A+ patient, HHV-6A was also found in liver and spleen (likely ciHHV-6A)
Sumiyoshi et al. (21)	B- and T-cell	PCR, SB	42/70 **(60%)**	55/56 (98.2%)	Reactive lymphadenopathy	NA	NA
Torelli et al. (23)	T- and B-cell	PCR	0/41 **(0%)**	NA	NA	NA	NA
Fox et al. (42)	SS-associated, B- and T-cell	SB	1/6 **(16.7%)**	NA	NA	NA	NA
Buchbinder et al. (43)	Various^1^	PCR, SB	20/23 **(87%)**	52/63 (83%)	Peripheral blood of AIDS patients	NA	Types of lymphoma and RH not differentiated in results
Josephs et al. (44)	B-cell	SB	3/>50 **(<6%)**	NA	NA	NA	NA
Jarrett et al. (24)	B- and T-cell	SB	2*/53 **(3.8%)**	0/35 (0%)	Non-lymphomatous tissues**	NA	*1 gastric DLBCL with SS, 1 TCL with preceding AITL **Reactive LNs, cancers, neoplasms, sarcoidosis, Sjögren's syndrome, miscellaneous
Daibata et al. (38)	MALT lymphoma	ISH	2/14 **(14.3%)**	NA	NA	NA	Positive cells randomly spread in the neoplastic tissues. Number of positive cells varied from case to case
Luppi et al. (26)	AITL^2^	IHC^a^	0/5 **(0%)**	0/16 (0%)	Non-neoplastic LNs^2^	NA	NA
Luppi et al. (26)	AITL^2^	IHC^b^	5/5 **(100%)**		Non-neoplastic LNs^2^	NA	Scattered plasma cells positive for p101K, gp106, and gp116. Isolated cells from controls positive for p101K, gp106, gp116
Luppi et al. (26)	Not specified^2^	IHC^a^^,^^b^	0/15 **(0%)**	0 (for p41)/16 (0%)	Non-neoplastic LNs^2^	NA	0 samples with neoplastic cells only were p101K+. Cytoplasmic staining observed for p101K in rare plasma cells among neoplastic cells and isolated stromal cells in the LN capsule/surrounding tissue. Isolated cells stained for gp106, gp116
Razzaque et al. (17)	T- and B-cell	IHC	2*/6 **(28.6%)**	NA	NA	NA	~30% of cells were positive for antigens *1 B-cell, 1 T-cell
Rojo et al. (28)	T- and B-cell	ISH	19/22 **(86.4%)**	13/13 (100%)	LNs from APL and RH	NA	By IHC, fewer cells (usually rare scattered lymphoid cells and monocytes expressing p41 and HAR) were positive for HHV-6 antigens in NHL cases than APL cases
Yin et al. (45)	Not specified	ISH	8/45 **(17.8%)**	NA	NA	NA	NA
Buchbinder et al. (43)	Various^1^	ISH	3/23 **(13%)**	NA	NA	NA	In positive samples, 1/10,000 cells was positive Lymphomas and RH not differentiated in results
Cao et al. (29)	Not specified	VirusScan	1/43 **(2.3%)**	NA	NA	NA	NA
Strong et al. (46)	Not specified	Next-generation RNA-sequencing, RNA CoMPASS	2/118 **(1.7%)**	NA	NA	0%, 100%; 50% EBV+	NA

*AIDS, acquired immune deficiency syndrome; AILD, angioimmunoblastic lymphadenopathy with dysproteinaemia; AITL, angioimmunoblastic T cell lymphoma; ALC, anaplastic large cell; APL, atypical polyclonal lymphoproliferation; CD, Castleman's disease; DLBCL, diffuse large B-cell lymphoma; HIV, human immunodeficiency virus; HL, Hodgkin lymphoma; HPS, hemophagocytic syndrome; IHC, immunohistochemistry; IL, immunoblastic lymphadenopathy; ISH, in situ hybridization; LN, lymph node; LP, lymphomatoid papulosis; MALT, mucosa-associated lymphoid tissue; MF, mycosis fungoides; NA, not available; NHL, non-Hodgkin lymphoma; NKTL, NK T cell lymphoma; PBLs, peripheral blood lymphocytes; PCLBCL, primary cutaneous large B-cell lymphoma; PEL, primary effusion lymphoma; PL, pityriasis lichenoides; PMLBCL, primary mediastinal large B-cell lymphoma; PTCL, peripheral T cell lymphoma; RH, reactive lymphoid hyperplasia; SB, southern blot; SNC, small noncleaved cell; SS, Sjogren's syndrome; TCL, T cell lymphoma; VirusScan, VirusScan bioinformatics system; WM, Waldenstrom's macroglobulinemia*.

^*^For those studies that employed both PCR and Southern Blot, only PCR results are presented.

^*^If quantitative data is unavailable for any given method or for controls, the data is excluded.

^*^Cao et al. (29) and Cantalupo et al. (30) both analyzed samples from the same database containing deep-sequencing reads from human tumors.

^*^IHC was performed using antibodies against

a p41 and

b *p101K, gp106, gp116*.

1 *HL (3), NHL (10), AILD (1), atypical RH (5), RH (4)*.

2 *Samples HHV-6 positive by PCR*.

*Bold values represent the percentage of HHV-6-positive samples among samples tested*.

By IHC/ISH, HHV-6 DNA and antigens have been detected in AITL, MALT lymphoma, unspecified NHL, and unspecified T- and B-cell lymphomas, but the signal was localized in scattered cells, often plasma cells, rather than neoplastic cells (17, 26, 28, 38, 43). RNA and DNA sequencing has identified HHV-6 in only up to 2.3% of NHL cases. On the whole, neither HHV-6A nor HHV-6B appear to be causative agents in NHL development, but investigation into specific subtypes of NHL, such as DLBCL, may be more constructive than viewing all types together. Moreover, the absence of viral DNA and antigens in neoplastic cells suggests that HHV-6 likely does not cause the development of most NHL, but it remains to be seen whether the virus might contribute to local immune dysfunction, or exacerbation of oncogenic effects of other agents, that predisposes to lymphomagenesis.

#### Leukemia (Table 3)

To date, the preponderance of data suggests no association between HHV-6 and leukemia. Contrasting findings, and differences in HHV-6 species predominating in bone marrow of leukemia patients, may stem from the use of different probes for HHV-6A and/or divergence in HHV-6A across geographical areas (50, 54). If this is the case, false negatives may have resulted. Likewise, PCR testing of blood samples has failed to demonstrate a strong association between HHV-6 viral load and leukemia (47, 52, 114, 115), and while serological testing has shown increased titers of HHV-6 antibody in leukemia (116–124), this is expected since HHV-6 reactivates in response to immunosuppression and chemotherapy (50). The inclusion of different subtypes of leukemia may result in skewed conclusions because HHV-6 may not have the same role across subtypes, as in the case of HL.

**Table 3:** Detection of HHV-6 in blood/bone marrow from patients with Leukemia.

**References**	**Type of leukemia in patient cohort, if specified**	**Detection method**	**HHV-6 positive patients/Total number of patients (%)**	**HHV-6 positive controls/Total number of controls (%)**	**Type of control tissue**	**Of typed samples: HHV-6A%, HHV-6B%, (HHV-6A/B Coinfection%); Coinfections with other viruses**	**Notes**
Nefzi et al. (47)	B-ALL	PCR	17*/20 **(85%)**	NA	NA	NA	*5 samples only positive at diagnosis, 3 positive at diagnosis and remission, 9 positive only at remission
Morales-Sánchez et al. (48)	66 B-cell, 4 T-cell ALL	PCR	6/70 **(9%)**	NA	NA	NA	BM samples
Gravel et al. (49)	Pediatric ALL	PCR	11*/293 **(3.8%)**	0/288 (0%)	NB	11%, 89% (all ciHHV-6A)	Detection threshold: ≥200 copies of HHV-6/μg DNA *10 pre-B, 1 pre-T
Faten et al. (50)	B-ALL	PCR	8/62 **(12.9%)**	NA	NA	0%, 100%	Median load in blood: 206 copies/million cells Median load in BM: 79 copies/million cells
Faten et al. (50)	T-ALL	PCR	0/7 **(0%)**	NA	NA	0%, 100%	NA
Faten et al. (50)	AML	PCR	4/64 **(6.3%)**	NA	NA	0%, 100%, (25% iciHHV-6B)	Median load in blood: 206 copies/million cells Median load in BM: 34 copies/million cells
Hubacek et al. (51)	Pediatric, 318 ALL, 21 AML	PCR	86/339 **(25.4%)**	NA	NA	NA	Community strains of HHV-6 only (not iciHHV-6)
Hubacek et al. (51)	Pediatric, 318 ALL, 21 AML	PCR	5/339 **(1.5%)**	NA	NA	80%, 20%	Testing for iciHHV-6 strains only
Seror et al. (52)	Pediatric B-ALL	PCR	16*/31 **(51.6%)**	NA	NA	0%, 100%	*3 samples only positive at diagnosis, 2 positive at diagnosis and remission, 11 positive only at remission
Seror et al. (52)	Pediatric T-ALL	PCR	1*/5 **(20%)**	NA	NA	0%, 100%	*Negative at diagnosis, positive at remission
Csire et al. (53)	MM	PCR	13/69 **(18.8%)**	4*/26 (15.4%)	MGUS and WM tissues	NA	NA
Hermouet et al. (54)	B-CLL	PCR	3*/21 **(14.3%)**	5/24 (20.8%)	NB and BM	0%, 100%	*Blood samples
Hermouet et al. (54)	B-ALL	PCR	1*/29 **(3.4%)**	5/24 (20.8%)	NB and BM	0%, 100%	*Blood sample
Hermouet et al. (54)	AML	PCR	1*/16 **(6.3%)**	5/24 (20.8%)	NB and BM	0%, 100%	*BM sample
Hermouet et al. (54)	B-CLL	PCR	11*/21 **(52.4%)**	0/24 (0%)	NB and BM	100%, 0%	*All positive were blood samples
Hermouet et al. (54)	B-ALL	PCR	10*/29 **(34.5%)**	0/24 (0%)	NB and BM	100%, 0%	*3 blood samples, 7 BM
Hermouet et al. (54)	AML	PCR	6*/16 **(37.5%)**	0/24 (0%)	NB and BM	100%, 0%	*All positive were BM samples
Luka et al. (55)	Pediatric T-ALL	PCR	14/16*(87.5%)	NA	BM	NA	NA
Luka et al. (55)	B-ALL	PCR	0/10 **(0%)**	NA	BM	NA	NA
Luka et al. (55)	CLL	PCR	0/4 **(0%)**	NA	BM	NA	NA
Krueger et al. (56)	CML*	IHC	19/36 **(54%)**	NA	NA	NA	*BM cells
Luka et al. (55)	T-ALL^1^	ISH	8/8 **(100%)**	NA	NA	NA	% Positive cells varied between 20 and 58%
Luka et al. (55)	T-ALL	IA (early antigen)	4/8 **(50%)**	NA	NA	NA	% Positive cells varied between 2 and 7%
Luka et al. (55)	T-ALL	IA (late antigen)	0/8 **(0%)**	NA	NA	NA	NA
Cantalupo et al. (30)	AML	Pickaxe	1/173 **(0.6%)**	NA	NA	NA	NA
Cao et al. (29)	Not specified	VirusScan	1/161 **(0.6%)**	NA	NA	NA	NA

*ALL, acute lymphocytic leukemia; AML, acute myelogenous leukemia; BM, bone marrow; CLL, chronic lymphocytic leukemia; CML, chronic myelogenous leukemia; IA, immunofluorescence assay; IHC, immunohistochemistry; ISH, in situ hybridization; MGUS, monoclonal gammopathy of unknown significance; NA, not available; NB, normal blood; Pickaxe, Pickaxe virus detection and discovery computational pipeline; VirusScan, VirusScan bioinformatics system; WM, Waldenstrom's macroglobulinemia*.

^*^If quantitative data is unavailable for any given method or for controls, the data is excluded.

^*^Cao et al. (29) and Cantalupo et al. (30) both analyzed samples from the same database containing deep-sequencing reads from human tumors.

^*^IHC was performed using antibodies against p41.

1 *Samples HHV-6 positive by PCR*.

*Bold values represent the percentage of HHV-6-positive samples among samples tested*.

Only one group has investigated HHV-6 antigen expression in leukemia, with intriguing results: HHV-6 early antigen p41 was detected in bone marrow cells—blasts and megakaryocytes—of 54% of 36 patients with chronic myelogenous leukemia (56). Since bone marrow biopsies from healthy individuals were not readily available, comparison between these two populations were not carried out. While this has been one of the strongest studies on HHV-6 and leukemia as it revealed active infection in approximately half of patients, standing alone, it is not enough to draw any clinically significant conclusions *per se*. Unfortunately, serological testing and PCR analysis of blood are inadequate in determining the role of HHV-6 in leukemia, and these methods fall short in differentiating between pathological and background HHV-6 infections. As blood is a relevant sample used in evaluating leukemia, this presents another hurdle; latent HHV-6 or HHV-6 undergoing low-level reactivation is occasionally present in healthy individuals. For these reasons, although many studies have made preliminary investigations into HHV-6 in several types of leukemia, forward progress of our understanding of the virus in these cancers has been stunted by the reliance upon blood PCR and serological analysis.

### Neurological (Table 4)

#### Glial tumors

Both HHV-6A and HHV-6B are neurotropic viruses, with glial cells being the most common viral reservoir in the nervous system. HHV-6B infection of the brain is associated with encephalitis and febrile seizures, while the presence of HHV-6A has been studied in the context of multiple sclerosis. Several studies have shown that HHV-6 infects human astrocytes and oligodendrocytes (125–128), and both species impair glutamate uptake (129) and induce chemokine/cytokine dysregulation in persistently infected glial cells (130). Long-term latent infection is also implicated in demyelination, especially in white matter (131).

**Table 4:** Detection of HHV-6 in tumor tissue from patients with cancers of the nervous system.

**References**	**Cancer type**	**Detection method**	**HHV-6 positive patients/Total number of patients (%)**	**HHV-6 positive controls/Total number of controls (%)**	**Type of control tissue**	**Of typed samples: HHV-6A%, HHV-6B%, (HHV-6A/B Coinfection%); Coinfections with other viruses**	**Notes**
Zheng et al. (57)	PA, non-invasive	PCR	7/30 **(23.3%)**	NA	NA	0%, 100%; 43% HPV+	Positive rate of HPV16 and HHV6-B infection positively correlated with PA invasiveness
Zheng et al. (57)	PA, invasive	PCR	16/30 **(53.3%)**	NA	NA	0%, 100%; 63% HPV+	Positive rate of HPV16 and HHV6-B infection positively correlated with PA invasiveness
Lin et al. (58)	Glioblastoma	ddPCR	6/39 **(15.4%)**	2/32 (6.2%)	Post-mortem BT without ND	0%, 100%	NA
Lin et al. (58)	Astrocytoma grade III, OCT-preserved	ddPCR	2/10 **(20%)**	2/32 (6.2%)	Post-mortem BT without ND	0%, 100%	NA
Chi et al. (59)	Astrocytoma	PCR	7/17 **(41.2%)**	1/13 (7.7%)	Post-mortem BT without ND	NA	NA
Chi et al. (59)	Glioblastoma multiforme	PCR	7/14 **(50%)**	1/13 (7.7%)	Post-mortem BT without ND	NA	NA
Chi et al. (59)	Oligodendroglioma	PCR	3/9 **(33.3%)**	1/13 (7.7%)	Post-mortem BT without ND	NA	NA
Crawford et al. (60)	Pediatric glioma	PCR (U57)	42/66 **(63.6%)**	7*/32 (22%)	Pediatric post-mortem BT without ND	74%, 26%	NA
Crawford et al. (60)	Pediatric glioma	PCR (U31)	47/66 **(71.2%)**	11*/32 (34%)	Pediatric post-mortem BT without ND	74%, 26%	*4/11 (33%) HHV-6A in controls
Crawford et al. (60)	Pediatric non-glial brain tumors^1^	PCR (U57)	26/54 **(48.1%)**	7/32 (22%)	Post-mortem BT without ND	67%, 33%	NA
Crawford et al. (60)	Pediatric non-glial brain tumors^1^	PCR (U31)	26/54 **(48.1%)**	11*/32 (34%)	Post-mortem BT without ND	67%, 33%	*4/11 (33%) HHV-6A in controls
Crawford et al. (61)	Adult CNS tumors*	PCR	14/30 **(47%)**	0/25 (0%)	Non-tumor BT, BT from other ND^2^	43%, 57%	*25 astrocytoma, 4 oligodendroglioma, 1 neuronal tumor
Neves et al. (62)	Pilocytic astrocytoma	PCR	0/35 **(0%)**	0/10 (0%)	Post-mortem cerebellum without ND	NA	NA
Cuomo et al. (63)	CNS tumors	PCR	43/115 **(37.3%)**	10*/31 (32.2%)	Post-mortem BT without ND	77%, 23%	*70% HHV-6A, 30% HHV-6B in controls
Chan et al. (64)	CNS tumors	PCR	8/98 **(8.2%)**	NA	NA	38.5%, 62.5%	NA
Luppi et al. (65)	Glial tumors	PCR	6/37 **(16.2%)**	10*/16 (62.5%)	Post-mortem BT without ND, BT from patients who died of AIDS	NA	*4/10 (57%) HIV+, 6/10 (66%) HIV-
Liedtke et al. (66)	Cerebral lymphoma	PCR	0/5 **(0%)**	3/20 (15%)	Post-mortem BT and spleen from cases without ND	NA	NA
Paulus et al. (67)	Primary cerebral lymphomas	PCR, SB	1/40 **(2.6%)**	NA	NA	NA	NA
Zheng et al. (57)	Pituitary adenoma, non-invasive	IHC^a^	7/30 **(23.3%)**	NA	NA	0%, 100%	NA
Zheng et al. (57)	Pituitary adenoma, invasive	IHC^a^	16/30 **(53.3%)**	NA	NA	0%, 100%	NA
Chi et al. (59)	Astrocytoma	IHC^b^	5/17 **(29.4%)**	0/13 (0%)	Post-mortem BT without ND	NA	Strong nuclear and cytoplasmic staining, 1/4 glioma cyst fluid samples was HHV-6+
Chi et al. (59)	Glioblastoma multiforme	IHC^b^	5/14 **(35.7%)**	0/13 (0%)	Post-mortem BT without ND	NA	Strong nuclear and cytoplasmic staining,1/4 glioma cyst fluid samples was HHV-6+
Chi et al. (59)	Oligodendroglioma	IHC^b^	3/9 **(33.3%)**	0/13 (0%)	Post-mortem BT without ND	NA	Strong nuclear and cytoplasmic staining, 1/4 glioma cyst fluid samples was HHV-6+
Crawford et al. (60)	Pediatric glioma	ISH	73/128 **(57%)**	10/32 (31%)	Pediatric post-mortem BT without ND	NA	More positivity in tumor tissue than adjacent normal BT
Crawford et al. (60)	Pediatric non-glial brain tumors	ISH	10/22 **(45%)**	10/32 (31%)	Pediatric post-mortem BT without ND	NA	More positivity in tumor tissue than adjacent normal BT
Crawford et al. (60)	Pediatric glioma	IHC^c^	48/102 **(47%)**	6/32 (18%)	Pediatric post-mortem BT without ND	NA	Staining of astrocytes. Increased positivity in glial compared to non-glial tumors. Significant difference in immunopositivity between lower and higher grade gliomas observed (42 and 19% positive, respectively)
Crawford et al. (60)	Pediatric non-glial brain tumors	IHC^c^	2/22 **(9%)**	6/32 (18%)	Pediatric post-mortem BT without ND	NA	NA
Crawford et al. (61)	CNS tumors	ISH	106/224 **(47%)**	0/25 (0%)	Non-tumor BT, BT from other ND^2^	NA	Perinuclear/cytoplasmic staining, predominantly in tumor, not vessels/non-tumor tissue
Crawford et al. (61)	Glial tumors	IHC^d^	62/217 **(29%)**	0/25 (0%)	Non-tumor BT, BT from other ND^2^	NA	Staining in cytosol, relatively heterogeneous distribution
Crawford et al. (61)	Glial tumors	IHC^c^	78/224 **(35%)**	0/25 (0%)	Non-tumor BT, BT from other ND^2^	NA	Staining in cytosol, relatively heterogeneous distribution
Crawford et al. (61)	Non-glial brain tumors	IHC^d^	6/60 **(10%)**	0/25 (0%)	Non-tumor BT, BT from other ND^2^	NA	Staining in cytosol, relatively heterogeneous distribution
Crawford et al. (61)	Non-glial brain tumors	IHC^c^	6/58 **(10%)**	0/25 (0%)	Non-tumor BT, BT from other ND^2^	NA	Staining in cytosol, relatively heterogeneous distribution
Cuomo et al. (63)	CNS tumors	IHC^d^	4/6 **(66.7%)**	0/7 (0%)	Post-mortem BT without ND (HHV-6 DNA+)	NA	Focal or scattered staining, nuclear or nucleo-cytoplasmic localization in neoplastic cells. Endothelial cells, not inflammatory cells, stained in glioblastoma tissues
Cantalupo et al. (30)	Glioblastoma	Pickaxe	2/162 **(1.2%)**	NA	NA	NA	NA
Cantalupo et al. (30)	Low grade glioma	Pickaxe	1/100 **(1%)**	NA	NA	NA	NA
Cao et al. (29)	Glioblastoma	VirusScan	1/169 **(0.59%)**	NA	NA	NA	NA
Cao et al. (29)	Low grade glioma	VirusScan	3/530 **(0.57%)**	NA	NA	NA	NA
Strong et al. (68)	Glioma	RNA sequencing	1/170 **(0.59%)**	0/5 (0%)	Normal BT	NA	NA
Duncan et al. (69)	Brain tumors*	Digital karyotyping	0/41 **(0%)**	NA	NA	NA	*Glioblastoma multiforme, astrocytoma, oligodendroglioma, medulloblastoma

*BM, bone marrow; BT, brain tissue; CNS, central nervous system; ddPCR, droplet digital PCR; HPV16, human papillomavirus 16; IHC, immunohistochemistry; ISH, in situ hybridization; IV, intravenous; NA, not available; ND, neurological diseases; PA, pituitary adenoma; PCR, polymerase chain reaction; Pickaxe, Pickaxe virus detection and discovery computational pipeline; VirusScan, VirusScan bioinformatics system*.

^*^If quantitative data is unavailable for any given method or for controls, the data is excluded.

^*^Cao et al. (29) and Cantalupo et al. (30) both analyzed samples from the same database containing deep-sequencing reads from human tumors.

^*^IHC was performed using

a *unnamed anti-HHV-6B antibody*,

b *unnamed anti-HHV-6 monoclonal antibody*,

c antibodies against gp116/64/54 and

d *p41*.

1 *Meningioma, medulloblastoma, germinoma*.

2 *Other ND listed: Stroke, Alzheimer's, Parkinson's, subarachnoid hemorrhage, contusion*.

*Bold values represent the percentage of HHV-6-positive samples among samples tested*.

One small study using droplet digital PCR (ddPCR) reported HHV-6B in 3 of 19 (15.8%) formalin-fixed paraffin embedded (FFPE) glioblastoma samples, 3 of 20 (15%) frozen glioblastoma samples, and 2 of 10 (20%) frozen astrocytoma grade III specimens. All were negative for cytomegalovirus (CMV) and HHV-6A (58). The viral loads of HHV-6B in tumors ranged from <100 to >20,000 copies/million cells. While the low viral loads likely indicate latency, the high loads suggest active infection, or potentially iciHHV-6. Of note, nested PCR is more sensitive, but also more prone to false positives, than ddPCR or sequencing analysis (132).

Analysis of 120 pediatric gliomas from 88 untreated patients using ISH, IHC, and nested PCR revealed higher rates of expression of the proteins U57 (major capsid protein) and U31 (large tegument protein) in tumors than in non-tumor controls (60). HHV-6 antigens were identified in 58% of low-grade gliomas compared to 19% of high-grade gliomas and 25% of non-glial tumors. The same group investigated the presence of HHV-6 in adult glial tumors and found that 47% of tumors tested positive for U57 compared to 0 of 25 controls (61). Moreover, HHV-6A early and HHV-6B late antigens were detected three times more frequently in glial tumors than in non-glial tumors, signifying more frequent active infection among those with glial tumors. Whether this correlation represents opportunistic reactivation due to the tumor will be an important avenue to explore.

These findings are supported by a more recent study, which analyzed 40 glioma tissue specimens and 13 normal brain tissue specimens (59). Using nested PCR, HHV-6 DNA was identified in 17 of 40 (42.5%) glioma tissue samples, compared to only 1 of 13 (7.7%) control samples. Along the same lines, 13 of 40 (32.5%) other glioma specimens stained positively for HHV-6, while no HHV-6 immunoreactivity was detected in normal brain tissues. Of note, HHV-6A was isolated from the glioma cyst fluids, and the HHV-6-infected specimens displayed higher levels of interleukin-6 (IL-6), IL-8, and transforming growth factor-β (TGF-β).

Taken together, the *in vitro* and *in vivo* data suggests that both species of HHV-6 are present in glioma. The greater prevalence of HHV-6 DNA and proteins among glial tumors compared to controls and the altered cytokine profile in HHV-6-positive specimens is suggestive of a potential role in gliomagenesis. To confirm this hypothesis, further studies are necessary to determine the localization of HHV-6 antigens, longitudinally investigating the effects of HHV-6 infection in animal models of glioma. Ultimately, a clinical trial is warranted to determine what role, if any HHV-6 plays in adult and pediatric glioma.

#### Pituitary adenomas

HHV-6B was recently implicated in the progression of invasive pituitary adenomas (PAs) through the toll-like receptor 3 (TLR3) signaling pathway. TLR3 is known to recognize double stranded RNA; however, studies have shown that the TLR3 cascade can also be activated by herpesviruses (133). Among 30 patients with invasive PAs and 30 patients with non-invasive PAs, HHV-6B DNA was detected in biopsy samples from 53.55% of invasive cases and 30% of non-invasive cases (57). Similarly, in invasive PA, TLR3 mRNA and protein were significantly higher than in noninvasive PA. TLR3 activity contributes to the innate immune response during viral infection via recognition of virus-associated double stranded RNA, but it has also been noted for its involvement in generating a pro-tumorigenic local environment and in promoting tumor cell invasion and proliferaation.

Rat pituitary adenoma G3 cells challenged with polyinosinic:polycytidylic acid (Poly(I:C)), a TLR3 agonist serving as a viral mimic, increased TLR3 expression and cell proliferation through the TLR3 signaling pathway (57). In addition, these cells expressed higher levels of Bcl-2, which can inhibit apoptosis, and lower levels of cleaved caspase 3. NF-κB, which is involved in cell proliferation, promotion of tumor invasion, and activation of inflammatory cytokines, was also activated, as were NF-κB-regulated genes for the inflammatory cytokines MMP9, IL-6, IL-1β, and TNF-α. Further studies on whether HHV-6 plays a direct role in TLR3 activation are necessary to confirm the significance of this association. This is the only study of its kind, but it suggests that perhaps the immunomodulatory capacity of HHV-6, and particularly its effects on innate immune effectors and mediators, leads to neurological dysfunction over time, as has recently been suggested in Alzheimer's disease (Readhead 2018), that may promote oncogenesis.

### Gastrointestinal (Table 5)

Gastrointestinal cancer (GIC) typically develops from a benign polyp, becomes an adenoma, and finally transforms into a carcinoma (134). One study detected HHV-6 in the GI tract of 63% of healthy individuals (135), whereas another study detected HHV-6 in 23% of liver transplant patients and 19% of immunocompetent patients (136). HHV-6 causes a range of GI symptoms, including diarrhea, colitis, and bacterial infection of the digestive tract (137), and an association between biliary complications and HHV-6 has been reported in many studies (136, 138).

**Table 5:** Detection of HHV-6 in tumor tissue from patients with gastrointestinal cancers.

**References**	**Cancer type**	**Detection method (ISH/IHC)**	**HHV-6 positive patients/Total number of patients (%)**	**HHV-6 positive controls/Total number of controls (%)**	**Type of control tissue**	**HHV-6 variant (A/B); Coinfections**
Halme et al. (70)	Colon adenoma	ISH	7/8 **(87.5%)**	0/3 (0%)	Normal mucosal biopsies	HHV-6B
Halme et al. (70)	Colon adenoma	IHC	5/8 **(62.5%)**	0/3 (0%)	Normal mucosal biopsies	HHV-6B; 3/8 total samples CMV+
Cao et al. (29)	Esophageal cancer	VirusScan	8/125 **(6.4%)**	NA	NA	NA
Cantalupo et al. (30)	Stomach cancer	Pickaxe	5/127 **(3.9%)**	0/16 (0%)	Adjacent normal tissue	NA
Cao et al. (29)	Stomach cancer	VirusScan	10/285 **(3.5%)**	NA	NA	NA
Cantalupo et al. (30)	Colon cancer	Pickaxe	19/407 **(4.7%)**	0/21 (0%)	Adjacent normal tissue	NA
Cao et al. (29)	Colon cancer	VirusScan	27/468 **(5.8%)**	NA	NA	NA
Cantalupo et al. (30)	Rectal cancer	Pickaxe	6/156 **(3.8%)**	1/5 (20%)	Adjacent normal tissue	NA
Cao et al. (29)	Rectal cancer	VirusScan	6/164 **(3.7%)**	NA	NA	NA
Cantalupo et al. (30)	Pancreatic cancer	Pickaxe	2/40 **(5%)**	0/1 (0%)	Adjacent normal tissue	NA
Cao et al. (29)	Pancreatic cancer	VirusScan	3/179 **(1.7%)**	NA	NA	NA
Duncan et al. (69)	Colorectal carcinomas	Digital karyotyping	0/3 **(0%)**	5/7 (71.4%)	Matched normal samples from individuals with colorectal liver metastases	NA
Duncan et al. (69)	Colorectal liver metastases	Digital karyotyping	3/7 **(42.9%)**	5/7 (71.4%)	Matched normal samples from the same individuals with colorectal liver metastases	NA
Cantalupo et al. (30)	Liver cancer	Pickaxe	0/68 **(0%)**	6/36 (16.7%)	Adjacent normal tissue	NA
Cao et al. (29)	Liver cancer	VirusScan	0/269 **(0%)**	NA	NA	NA

*IHC, immunohistochemistry; ISH, in situ hybridization; NA, not available; Pickaxe, Pickaxe virus detection and discovery computational pipeline; VirusScan, VirusScan bioinformatics system*.

^*^If quantitative data is unavailable for any given method or for controls, the data is excluded.

^*^Cao et al. (29) and Cantalupo et al. (30) both analyzed samples from the same database containing deep-sequencing reads from human tumors.

*Bold values represent the percentage of HHV-6-positive samples among samples tested*.

Using a bioinformatics and sequencing approach to investigate the viral basis of 6,813 tumors and 559 adjacent normal samples, Cao et al. found HHV-6 at a relatively high prevalence in GICs (29). Specifically, HHV-6 was detected in 26 cases of colon adenocarcinomas (5.8% of total cases), 9 cases of stomach adenocarcinomas (3.5%), 7 cases of esophageal cancer (6.4%), and 6 cases of rectal adenocarcinomas (3.7%). The virus was not detected in paired adjacent normal samples. Cantalupo et al. analyzed tumor sample sequences from The Cancer Genome Atlas (TCGA) as well and identified HHV-6 sequences in 3.9% of stomach cancer samples and 4.7% of colon cancers. In contrast, the virus was neither found in 16 paired normal stomach samples nor in 21 normal colon samples (30). Rectal cancer samples were positive at a comparable frequency (3.8%), but 1 of 5 normal controls was also positive. EBV and CMV were also frequently detected in GIC, and together with HHV-6, they were the most commonly identified viruses in stomach and colorectal tumors. In some cases, HHV-6 was detected by both DNA-sequencing and RNA-sequencing. This sequencing approach has a low risk of false-positives.

In addition, HHV-6B was frequently detected in adenomatous polyps of the colon, while mucosal biopsies from patients without adenomas revealed neither IHC nor ISH positivity (70). Gastrointestinal cancers, in addition to oral cancer, brain tumors (particularly glial tumors), and NSHL represent malignancies that deserve priority for future studies on the oncogenic capacity of HHV-6A and HHV-6B.

### Gynecological (Table 6)

#### Ovarian cancer

HHV-6A also emerged as a pathogen of interest in ovarian cancer in view of a broad-scale investigation of the ovarian cancer oncobiome using the microarray system PathoChip, which identified species of bacteria, fungi, parasites, and viruses that predominated in cancer tissue when compared to control tissue (71). Conserved and specific probes for both HHV-6A and−6B were found in the cancer biopsies but were absent in the 20 matched ovarian tissue samples and 20 non-matched ovarian biopsies. Ten instances of HHV-6A integration in various chromosomes were detected, with U47, encoding envelope glycoprotein O, identified as the most commonly inserted viral sequence. Most integrations were found in intronic or intergenic regions, but some were present at exonic and sub-telomeric loci. Of the genes that HHV-6A sequences were found to be integrated into or located near, most were significantly associated with cancers, and six, including CPLX1, IGFBP3, and the oncogene SH3RF2, were associated with malignant tumor formation (*p* = 8.45 × 10^−7^). Although HHV-6A was one of many pathogens detected more frequently in cancer tissues than in matched controls, the association between the genes that the virus integrated into or near to and the development of cancer, as well as the lack of HHV-6 in non-cancerous samples, makes a case for closer examination of integrated HHV-6 in ovarian cancer. A single study is insufficient to draw strong conclusions from, so reproduction of these initial findings is a logical next step.

**Table 6:** Detection of HHV-6 in tumor tissue from patients with gynecological cancers.

**References**	**Cancer type**	**Detection method**	**HHV-6 positive patients/Total number of patients (%)**	**HHV-6 positive controls/Total number of controls (%)**	**Type of control tissue**	**Of typed samples: HHV-6A%, HHV-6B%, (HHV-6A/B Coinfection%); Coinfections with other viruses**
Banerjee et al. (71)	Ovarian cancer	PathoChip, next-generation sequencing	16/99 **(16.2%)**	0/40 (0%)	20 matched, 20 unmatched ovarian tissues	63%, 37%
Cantalupo et al. (30)	Ovarian cancer	Pickaxe	0/93 **(0%)**	NA	NA	NA
Duncan et al. (69)	Ovarian carcinoma	Digital karyotyping	0/7 **(0%)**	NA	NA	NA
Cantalupo et al. (30)	Uterine carcinosarcoma	Pickaxe	1/95 **(1%)**	0/5 (0%)	Adjacent normal tissue	NA
Cao et al. (29)	Uterine carcinosarcoma	VirusScan	0/57 **(0%)**	NA	NA	NA
Cantalupo et al. (30)	Cervical SCC, endocervical adenocarcinoma	Pickaxe	2/255 **(0.8%)**	1/208 (0.5%)	Adjacent normal tissue	NA
Cao et al. (29)	Cervical SCC, endocervical adenocarcinoma	VirusScan	2/252 **(0.8%)**	NA	NA	NA
Broccolo et al. (72)	Cervical neoplasia^1^	PCR	39/103 **(37.9%)**	9/66 (14%)	Normal CT	22/23 with high grade dysplasia had high-risk HPV
Tran-Thanh et al. (73)	Cervical neoplasia^2^	PCR	6/195 **(3.1%)**	1/125 (0.8%)	Normal CT	86% HPV+
Chan et al. (74)	Cervical neoplasia^2^	PCR	7/187 **(3.7%)**	7/201 (3.5%)	CT, normal or inflamed	57% HPV+
Romano et al. (75)	Cervical neoplasia^2^	PCR	1/51 **(2%)**	0/58 (0%)	CT, normal or inflamed	0%, 100%; HPV+
Wang et al. (76)	Cervical neoplasia	PCR	2/8 **(25%)**	NA	NA	50% 50%
Chen et al. (77)	Cervical carcinoma, intraepithelial neoplasia	PCR, SB, ISH	6/72 **(18.8%)**	0/30 (0%)	CT, normal and cervicitis	66.7% HPV+
Arivananthan et al. (78)	Cervical carcinoma	ISH	10/30 **(33.3%)**	0/7 (0%)	Normal oral, salivary gland tissues, CT	20%, 50%, (30%)
Yadav et al. (79)	Cervical neoplasia	ISH	10/26 **(38.5%)**	1/8 (12.5%)	Normal CT	28.6%, 28.6%, (42.8%)
Yadav et al. (79)	Cervical neoplasia	IHC	14^3^/26 **(53.8%)**	5/8 (62.5%)	Normal CT	NA

*CT, cervical tissue; HGSIL, high grade squamous intraepithelial lesion; IHC, immunohistochemistry; HPV, human papillomavirus; ISH, in situ hybridization; LGSIL, low grade squamous intraepithelial lesion; NA, not available; Pickaxe, Pickaxe virus detection and discovery computational pipeline; SB, southern blot; SCC, squamous cell carcinoma; VirusScan, VirusScan bioinformatics system*.

^*^For studies that employed both PCR and SB or ISH, only PCR results are presented.

^*^If quantitative data is unavailable for any given method or for controls, the data is excluded.

^*^Cao et al. (29) and Cantalupo et al. (30) both analyzed samples from the same database containing deep-sequencing reads from human tumors.

^*^IHC was performed using antibodies against gp116/64/54 and p41/38.

1 *LGSIL, HGSIL*.

2 *LGSIL, HGSIL, SCC, adenocarcinoma*.

3 *Staining in transformed cells. In normal tissue, staining in endocervical ciliated columnar-epithelial cells, some cells in subepithelial mucosa*.

*Bold values represent the percentage of HHV-6-positive samples among samples tested*.

### Head and neck (Table 7)

Oral cancer is often linked to tobacco and/or alcohol use, as well as to HPV infections. The salivary glands serve as a reservoir for HHV-6, which is persistently shed in the saliva. While HHV-6 has not been shown to cause oral cancer, by facilitating the transformation process, HHV-6 may serve as a co-factor with HPV and chemical carcinogens found in tobacco and alcohol.

**Table 7:** Detection of HHV-6 in tumor tissue from patients with head and neck cancers.

**References**	**Cancer type**	**Detection method**	**HHV-6 positive patients/Total number of patients (%)**	**HHV-6 positive controls/Total number of controls (%)**	**Type of control tissue**	**Of typed samples: HHV-6A%, HHV-6B%, (HHV-6A/B Coinfection%); Coinfections with other viruses**	**Notes**
Cantalupo et al. (30)	Head/neck squamous cell carcinoma	Pickaxe	11/517 **(2.1%)**	6/515 (1.2%)	Adjacent normal tissue	NA	NA
Cao et al. (29)	Head/neck squamous cell carcinoma	VirusScan	12/498 **(2.4%)**	NA	NA	NA	NA
Arivananthan et al. (78)	Laryngeal	ISH	4/4 **(100%)**	0/7 (0%)	Normal oral, cervical, salivary gland tissues	0%, 75%, (25%)	NA
Arivananthan et al. (78)	Salivary gland	ISH	6/8 **(75%)**	0/7 (0%)	Normal oral, cervical, salivary gland tissues	0%, 50%, (50%)	NA
Arivananthan et al. (78)	Oral SCC	ISH	16/21 **(76.2%)**	0/7 (0%)	Normal oral, cervical, salivary gland tissues	13%, 31%, (56%)	NA
Yadav et al. (80)	Oral SCC	ISH	33/42 **(78.6%)**	12/30 (40%)	Normal mucosa, LP, leukoplakia	9%, 42%, (48%)	NA
Yadav et al. (80)	Oral SCC	IHC	41/51 **(80.4%)**	15/25 (60%)	Normal mucosa, LP, leukoplakia	NA	Cytoplasmic and nuclear staining in some cells. 60% of 15 screened SCC expressed only a limited number of antigens
Yadav et al. (81)	Oral SCC	IHC	7/7 **(100%)**	0/3 (0%)	NPC	NA	Cytoplasmic staining of transformed SCC cells, sometimes also strong positivity in the membrane and nucleus of squamous cells
Saravani et al. (82)	Oral SCC	PCR	13/45 **(28.9%)**	NA	NA	NA	No correlation between mean viral load and SCC grade
Yadav et al. (80)	Oral SCC	PCR	19/24 **(79%)**	5/35 (14.3%)	Normal mucosa, LP, leukoplakia, breast cancer	NA	NA
Yadav et al. (81)	Oral SCC	PCR	11/16 **(68.8%)**	NA	NA	NA	NA
Shanehsazzadeh et al. (83)	Salivary gland cancer*	PCR	28/60 **(46.7%)**	NA	NA	64% HHV-7+	*Pleomorphic and monomorphic adenoma, mucoepidermoid carcinoma
Chen et al. (84)	NPC	PCR, ISH	13/42 **(30.9%)**	1*/48 (2.1%*)	Nasopharynx, precancerous and normal	95% EBV+	*precancerous sample
Kositanont et al. (85)	NPC	PCR	5/34 **(14.7%)**	0/5 (0%)	Non-cancerous tissue	94% EBV+	NA

*BCC, basal-cell carcinoma; EBV, Epstein-Barr virus; HHV-7, human herpesvirus 7; IHC, immunohistochemistry; ISH, in situ hybridization; LP, lichen planus; NA, not available; NPC, nasopharyngeal carcinoma; Pickaxe, Pickaxe virus detection and discovery computational pipeline; SCC, squamous cell carcinoma; VirusScan, VirusScan bioinformatics system*.

^*^For studies that employed both PCR and ISH, only PCR results are presented.

^*^If quantitative data is unavailable for any given method or for controls, the data is excluded.

^*^Cao et al. (29) and Cantalupo et al. (30) both analyzed samples from the same database containing deep-sequencing reads from human tumors.

^*^IHC was performed using antibodies against gp116/64/54.

*Bold values represent the percentage of HHV-6-positive samples among samples tested*.

Both HHV-6 DNA and antigens were detected in early studies using tissue samples from oral mucosal tumors (80, 81), but they were undetectable in controls. Antigen (HHV-6 glycoprotein) was detected by immunoperoxidase staining in transformed squamous cells of all samples, where it was localized in the cytoplasm, cell membrane, and nucleus; it was not found in cells surrounding the carcinomas. Of seven HHV-6 antigen-positive tumor samples, only five were positive for HHV-6 DNA, suggesting relatively low levels of HHV-6 DNA within these cells (81). The failure to detect DNA in the antigen-expressing cells could also mean that the virus acts using a “hit-and-run” method, in which it participates in the early phases of tumor development by spurring oncogenic changes within the cell, but then effectively disappears (139). Additionally, elevated levels of IgA antibodies to HHV-6 were detected in five patients with advanced oral carcinoma, but in none of the controls.

Follow up investigations revealed HHV-6 DNA and antigens by PCR, ISH, and IHC in oral squamous cell, laryngeal, and salivary gland carcinomas but not in normal oral and salivary gland biopsies (78, 80). Many HHV-6-positive carcinoma samples revealed HHV-6A/HHV-6B co-infection. Most recently, HHV-6 DNA was found in 27% of FFPE tissue samples from oral squamous cell carcinomas (82). No association with mean viral load or histopathologic grade was identified. The level of contrast in HHV-6 DNA and proteins between oral cancer and control tissues is intriguing and merits further study. The presence of the virus in transformed cells- and not in cells outside of the tumor- is of particular consequence, and efforts should be made to replicate this finding with larger sample sizes. As only a single report on HHV-6 in oral cancer has been published since the turn of the century, renewed efforts are due to detect the presence of the virus- active or latent- in tumor cells.

### Weak associations

Qualitative PCR performed on non-melanoma skin cancer specimens revealed that 32% of tumors were HHV-6 positive as compared to 15% of healthy skin specimens (140), and Cao et al. found HHV-6 in only 0.4% of melanomas via sequencing (29). The current data, though very limited in scope, does not favor the involvement of HHV-6 in skin cancers and points instead to opportunistic reactivation. A similar situation is seen in such heterogeneous conditions as multiple myeloma, sarcoma, and prostate, uterine, liver, kidney, thyroid, and pancreatic cancers, for which few studies have been carried out, and associations are weak and not supportive of an etiological role for HHV-6.

Several studies indicate that HHV-6 does not contribute to breast cancer (78, 80, 141). Interestingly, the HHV-6B U54 tegument protein inhibits MCF-7 breast cancer cell proliferation *in vitro* by inhibiting NFAT activity (142).

## HHV-6 coinfections

HHV-6 has been detected in conjunction with several other pathogens in a range of conditions. Describing each such study is beyond the scope of this review, so only those studies providing the most meaningful data, in terms of their contributions to an understanding of cooperative activity between HHV-6 and other viruses, will be detailed.

### HHV-8 in NHL (Table 2)

HHV-6 DNA has been detected in many cases of B- and T-cell NHL (24, 44), sometimes in addition to other viruses. Nakayama-Ichiyama et al. (88, 89) reported dual HHV-6/HHV-8 infection in 2 cases, a primary cutaneous large B cell lymphoma and a diffuse large B-cell lymphoma (DLBCL) with dual HHV-6/HHV-8 positive, EBV-negative tumor cells, and negative serology for HIV and human T lymphotropic virus 1 (HTLV-1). The co-localization of these viruses in tumor cells of B-cell lymphomas is quite compelling and deserves follow-up. In a study of 191 primary effusion lymphoma (PEL) cases, HHV-6 was detected in 18% of HHV-8-negative tumors and in 5% of HHV-8-positive tumors (37). Since all PEL tumors are currently defined as HHV-8-positive, the diagnosis of PEL is questionable in some of these cases. HHV-6 infection of the HHV-8 positive B-cell lymphoma line BCBL-1 induces HHV-8 lytic activation (143), and it has been proposed that the simultaneous activity of an HHV-6 gene homologous to the adeno-associated virus (AAV) type 2 rep gene (144) and HHV-8 regulatory genes could play a role in lymphomagenesis (145, 146).

### EBV

#### NHL (Table 2)

B-cell (38, 46) and T-cell (35, 41) lymphomas with dual HHV-6 and EBV positivity have also been described, often using PCR. Standing alone, these PCR-based studies have not provided overly compelling evidence for or against a cooperative relationship in lymphomagenesis. In a recent case-control study of 214 NHL cases and 214 matched controls from three population-based prospective cohorts, seropositivity for HHV-6A immediate-early 1 protein (IE1A), but not HHV-6B IE1, was significantly associated with a greater risk of developing NHL of any type (OR 1.85, 95% CI 1.04–3.29) (147). However, levels of antibodies against EBV proteins ZEBRA and EA-D were slightly correlated with IE1A seropositivity, and the correlation between HHV-6A IE1A and NHL development was somewhat weaker after adjusting for these EBV-specific antibodies, suggesting that HHV-6A activity may interact with EBV and increase the risk of NHL. Alternatively, an immunosuppressive or otherwise altered environment in individuals at risk for NHL may set the stage for HHV-6A reactivation, although the finding that HHV-6B IE1 was not correlated with NHL argues against this. However, if this is the case, HHV-6A activity may serve as a biomarker that can be a predictive factor for NHL. The longitudinal nature of this study, the differentiation between HHV-6A and HHV-6B, and the use of a newly developed serological assay that can identify active HHV-6 were definite strengths. Subtype-specific analysis would be a worthwhile avenue to pursue going forward.

Among lymphomas demonstrating coinfection, the potential of a cooperative role has been most thoroughly described in the setting of angioimmunoblastic T-cell lymphoma (AITL). EBV and HHV-6 have been detected in B cells and plasma cells, respectively, of patients with AITL; neither virus was detected in these patients' neoplastic CD4^+^ T cells (35, 41). Among a cohort of EBV-positive AITL cases with advanced pattern III histology, some were HHV-6B-positive, whereas none of the dual-infected cases were typed as early pattern I histology (33). While the presence of both HHV-6 and EBV may indicate a cooperative pathogenic role, it is also possible that it merely indicates a level of immune dysfunction that allows for viral reactivation from latency. Interestingly, however, HHV-6 and EBV viral loads in the dual-positive tumors displayed an inverse relationship (i.e., samples with the highest EBV or HHV-6 load had relatively low levels of the other virus) (33). Early studies also reported evidence of HHV-6 infection in HIV-associated non-Hodgkin lymphoma (NHL). In this immunocompromised population, HHV-6 was found in up to 32% of B-cell lymphomas (16, 18, 19).

HHV-6 has been isolated from NHL biopsies at highly variable rates, and the wide range of detection methods used on neoplastic tissues, as well as the difficulty in obtaining healthy lymph nodes for use as controls, limits the ability to unequivocally confirm the involvement of HHV-6 in triggering malignant transformation in NHL. However, in the face of such ambiguity, comparison of other relevant factors can be helpful in determining how HHV-6 affects the development of NHL. For example, Zhou et al. found that HHV-6B viral load was dramatically higher in AITL tissue with pattern III histology (median 40 copies/10^3^ cells) than in samples with pattern II histology (median 0.7 copies/10^3^ cells) (33). While a high rate of AITL tissue samples were positive for HHV-6, the virus was not detected within malignant T cells; instead, infection of plasma cells was apparent in these samples (26). These results suggest that HHV-6-infected plasma cells may thrive in the immunosuppressive tumor microenvironment. Similarly, EBV-infected large B cells are typically present within AITL tumor tissue. The presence of these two herpesviruses (HHV-6 and EBV) in non-malignant cells within the tumor lends credence to the notion that these viruses are opportunistic pathogens that thrive in the immunosuppressive tumor microenvironment. Nevertheless, a secondary supportive or contributory role for these viruses in tumor growth cannot be ruled out.

#### Nasopharyngeal (Table 7)

A trend of HHV-6/EBV coinfection has been observed in EBV-associated nasopharyngeal carcinoma (NPC). Among 34 NPC tumor samples tested by PCR, all of which were either World Health Organization type 2 or 3, 14.7 and 94.1% were positive for HHV-6 and EBV DNA, respectively, while none of the nasopharyngeal tissue samples from five controls were positive (85). HHV-6 infection of NPC specimens has also been correlated with heightened expression of EBV LMP-1 (84). Whether this phenomenon is a result of HHV-6 activity or whether HHV-6 reactivation results from the effects of NPC remains to be determined. The difference in prevalence between HHV-6 and EBV suggests that, if HHV-6 is involved in some cases of NPC, it is involved in a more limited subset, and likely is not sufficient for transformation.

#### Pulmonary

Neoplastic cells isolated from pulmonary adenocarcinomas that contained HHV-6 by PCR (25%) were all co-infected with EBV (148). Cantalupo et al. identified HHV-6 DNA and/or RNA sequences in a small number (3.5%) of lung tumors and paired normal lung tissue (30). HHV-6 is likely not a primary carcinogenic agent in lung cancer, but its detection in some tumor cells supports a possibly contributory role.

### HPV

#### Cervical (Table 6)

There are over 100 types of HPV, of which at least 13 are important in the etiology of cervical cancer. HHV-6 has been shown to productively infect HPV-immortalized cells, transactivate HPV gene expression of oncoproteins, and enhance the expression of HPV RNA (75, 76, 149). Notably, two HHV-6 clones that were previously found to upregulate the expression of HPV-transforming genes were also found to transactivate the long terminal repeat of HIV-1 when combined with the HIV-1 transactivator TAT-1 in cervical carcinoma cell lines (150).

In an early analysis of squamous cell carcinoma (SCC) and cervical intraepithelial neoplasia, HHV-6A and/or B were detected by PCR in 8% of neoplastic cases compared to 0% of negative controls (77). In another early study, HHV-6 was detected by IHC in 54% of cervical carcinoma samples vs. 63% of normal tissues. However, in HHV-6+ carcinomas, virus was present within tumor cells, raising the possibility of differential viral activity across individuals through which HHV-6 may be relatively common in healthy cervical tissues, but may act as a contributory factor in cervical carcinogenesis in predisposed individuals. Of note, while HHV-6B was found in both cancerous and control samples, HHV-6A was only found in the cancerous tissues (79). Recent literature has implicated HHV-6A in cases of unexplained female infertility (151) and tissue taken from infertile women with endometrial HHV-6A infections has revealed an altered immune profile that points to local immune dysregulation in response to infection. HHV-6B has been found in the semen of healthy sperm donors as well as in the vaginal canal of healthy women (152, 153). It is possible that HHV-6A may be more pathogenic than HHV-6B in the female reproductive tract.

The presence of HHV-6 in HPV-infected women has also been correlated with a higher grade of squamous intraepithelial lesions; while 41% of high-grade lesions were HHV-6+, none of the normal HPV+ cervixes were HHV-6+ (72). A previous cohort, however, did not express this pattern, and although HHV-6 was found in 8.3% (2/24 samples) of cervical carcinomas, no carcinoma was coinfected with HPV (74).

#### Urological

Conflicting PCR results have been presented on the presence of HHV-6 in bladder cancer specimens. In one cohort, 7% of cases were HHV-6B+, compared to 0% of non-malignant bladder tissue. Of the HHV-6B+ positive cases, 40% were HPV co-infected (154). In another study, HHV-6 was found in both tumor and normal bladder tissues in equal proportion, and HPV was not detected in any sample (155). At present, these results are too limited in scope to draw firm conclusions but point to a bystander role for HHV-6.

## Inherited chromosomally integrated HHV-6

It has been suggested that iciHHV-6 might predispose the formation of marker chromosomes. In the case of a patient with DLBCL and iciHHV-6 integration in chromosome 17p, 10% of the metaphases analyzed contained a clone with a second HHV-6 signal in an extra marker chromosome (86) (Table 8). The authors pointed out that structurally abnormal chromosomes are commonly found in DLBCL, and that the high expression of HHV-6 U94, a protein with DNA-binding, exonuclease, and helicase-ATP activities, may have been involved in tumorigenesis.

**Table 8:** HHV-6 detection in individual cancer patients.

**References**	**Cancer type**	**Detection methods**	**HHV-6 positive tissues**	**HHV-6 cellular localization**	**Antigen detection**	**HHV-6 variant**	**Disease course**
Campioni et al. (86)	DLBCL	PCR, RT-PCR, FISH	Bone marrow, pleural effusion derived mesothelial cells, peripheral blood	17p chromosome region of a marker chromosome	U94 exhibited levels of transcription (100 ± 15 transcripts/1 μg RNA)	iciHHV-6A	An 82-years-old man with DLBCL died of respiratory insufficiency
Zhang et al. (87)	PEL-like lymphoma	PCR, FISH, ddPCR	Tumor tissue, peripheral blood	19q telomere absent from lymphoma cells despite retention of both copies of chromosome 19	NA	iciHHV-6A	A 73-years-old woman developed an HHV-8-unrelated PEL-like lymphoma that resolved following R-CHOP therapy, and she remained in remission for 9 years, eventually dying from complications of diabetes
Nakayama-Ichiyama et al. (88)	PCLBCL	IHC	Tumor tissue of the leg (HHV-8 coinfection)	Nucleoli of lymphoma cells	NA	NA	A 91-years-old woman developed PCLBCL, leg type. Lymphadenopathy disappeared and skin lesions showed improvement after R-CHOP therapy
Nakayama-Ichiyama et al. (89)	DLBCL	IHC	Tumor tissue of the leg (HHV-8 coinfection)	Nucleoli of lymphoma cells	NA	NA	A 69-years-old woman developed DLBCL, not otherwise specified (NOS), nongerminal center B-cell–like subtype, stage IIIA. Symptoms and lesions disappeared after treatment with rituximab, cyclophosphamide, doxorubicin, vincristine, and prednisolone
Lohi et al. (90)	ALL	PCR	Blood	22q-tel chromosome region	NA	iciHHV-6	A 3.5-years old girl was diagnosed with ALL. High-copy numbers of HHV-6 were found during induction therapy
Hubacek et al. (91)	AML	PCR, FISH	Hair follicles, blood, all sampled tissues	Integrated on a marker chromosome of unknown origin	NA	iciHHV-6A	A 33-years old woman with AML was found to have high levels of HHV-6A DNA in blood during treatment. She died of CMV pneumonitis after HSCT
Hubacek et al. (91)	ALL	PCR, FISH	Hair follicles, blood, all sampled tissues	18p11.3 chromosome region	NA	iciHHV-6B	A 6-years old boy with ALL developed GvHD post-transplant and later died of CMV pneumonitis
Lin et al. (92)	TCL	Viral culture	Bone marrow	NA	NA	NA	A 48-years-old man developed gammadelta T-cell lymphoma 4 years after undergoing kidney transplantation. Long term remission was not achieved
Daibata et al. (93)	Pre-B-cell ALL	PCR, SB, ISH, FISH	Liver, spleen, lungs, and brain, PBMC	1q44 chromosome region Nuclei of 80% of PBMCs and the majority of leukemic cells	NA	iciHHV-6B	An 83-years-old woman developed pre-B-cell ALL with a t(9;22)(q24; q11) chromosomal abnormality resulting in generation of Philadelphia (Ph1) chromosome
Daibata et al. (94)	AML and AILD	PCR	Lymph node, pericardial effusion, liver, BM, lungs, spleen, kidneys, heart	NA	NA	HHV-6B	A 47-years-old man with AML was diagnosed with AILD 4 months after induction of chemotherapy and died of fulminant hepatitis
Bandobashi et al. (95)	Burkitt's Lymphoma	PCR	Bone marrow	Lymphoma cells	NA	NA	A 58-years-old woman developed Burkitt's lymphoma. Chemotherapeutic response was minimal, the disease progressed into a leukemic phase, and she died of septicemia 6 weeks after admission
Maeda et al. (96)	NSHL	ISH, IHC, Serology	Lymph nodes	Macrophages and lymphocytes, predominantly in lymphoid follicles, but not in RS nor Hodgkin cells	Antigens detected in lymph nodes	NA	A 7-years-old boy showed changes developed HL of the NS-LD subtype. Chemotherapy resulted in complete remission
Stödberg et al. (97)	Diffuse leptomeningeal oligodendrogliomatosis	PCR	CSF, serum, and tumor tissue	NA	Limited attempts at antigen detection were unsuccessful	HHV-6A	A 2-years-old boy developed a leptomeningeal tumor. After chemotherapy and complementary antiviral therapy, the boy's clinical condition stabilized, and his predominant problem became a gradually more evident autistic disorder
Rantala et al. (98)	Pilocytic astrocytoma	PCR	CSF initially, tumor tissue	NA	NA	NA	A 14-months old girl developed a pilocytic astrocytoma in the cerebellum 11 months after a period of fever, exanthema, encephalitis, carditis, and intractable seizures, during which time HHV-6 was identified in the CSF. After removal of the tumor, she still had severe hypotonia, poor social contact, and up to 5 series of infantile spasms daily

*AILD, angioimmunoblastic lymphadenopathy with dysproteinaemia (also known as AITL); ALL, acute lymphocytic leukemia; AML, acute myeloblastic leukemia; CMV, cytomegalovirus; CSF, cerebrospinal fluid; ddPCR, droplet digital PCR; DLBCL, diffuse large B-cell lymphoma; FISH, fluorescence in situ hybridization; GvHD, graft vs. host disease; HL, Hodgkin's lymphoma; IHC, immunohistochemistry; ISH, in situ hybridization; NA, not available; NHL, non-Hodgkin lymphoma; NSHL, nodular sclerosis Hodgkin lymphoma; NS-LD, nodular sclerosis lymphocyte depleted; PBMCs, peripheral blood mononuclear cells; PCLBCL, primary cutaneous large B-cell lymphoma; PEL, primary effusion lymphoma; RS, Reed-Sternberg; RT-PCR, reverse transcriptase polymerase chain reaction; TCL, T cell lymphoma*.

Recently, iciHHV-6 was found in 2 of 35 pediatric adrenocortical tumors, and fluorescence *in situ* hybridization (FISH) revealed that the HHV-6 sequences were integrated at the telomeric region of 11p15, a site that is commonly implicated in this condition (156). In both cases, loss of heterozygosity (LOH) of chromosome 11 and heightened expression of IGF2 were demonstrated, as was paternal transmission of the integrated virus. LOH of chromosome 11p, via duplication of the paternal chromosome and loss of the maternal chromosome, as well as LOH at chromosome 17 appear to be early markers of adrenocortical carcinoma progression (157, 158). Integration of HHV-6 can also occur at chromosome 17p13 (159, 160), a locus that contains the TP53 tumor suppressor gene.

On the other end of the spectrum, a single report has documented the loss of iciHHV-6A from the telomere of chromosome 19q in an HHV-8-negative PEL-like lymphoma (87). Notably, loss of the integrated HHV-6A appeared to have occurred in the early stages of lymphoma development, raising the possibility of HHV-6A involvement in early chromosomal dysfunction. The unique nature of iciHHV-6 among human herpesviruses is an area deserving of greater investigation, and the effects of integration on chromosomal stability may be among the most intriguing subtopics in this field.

## Potential mechanisms of HHV-6-associated neoplasia

**Figure 2:**
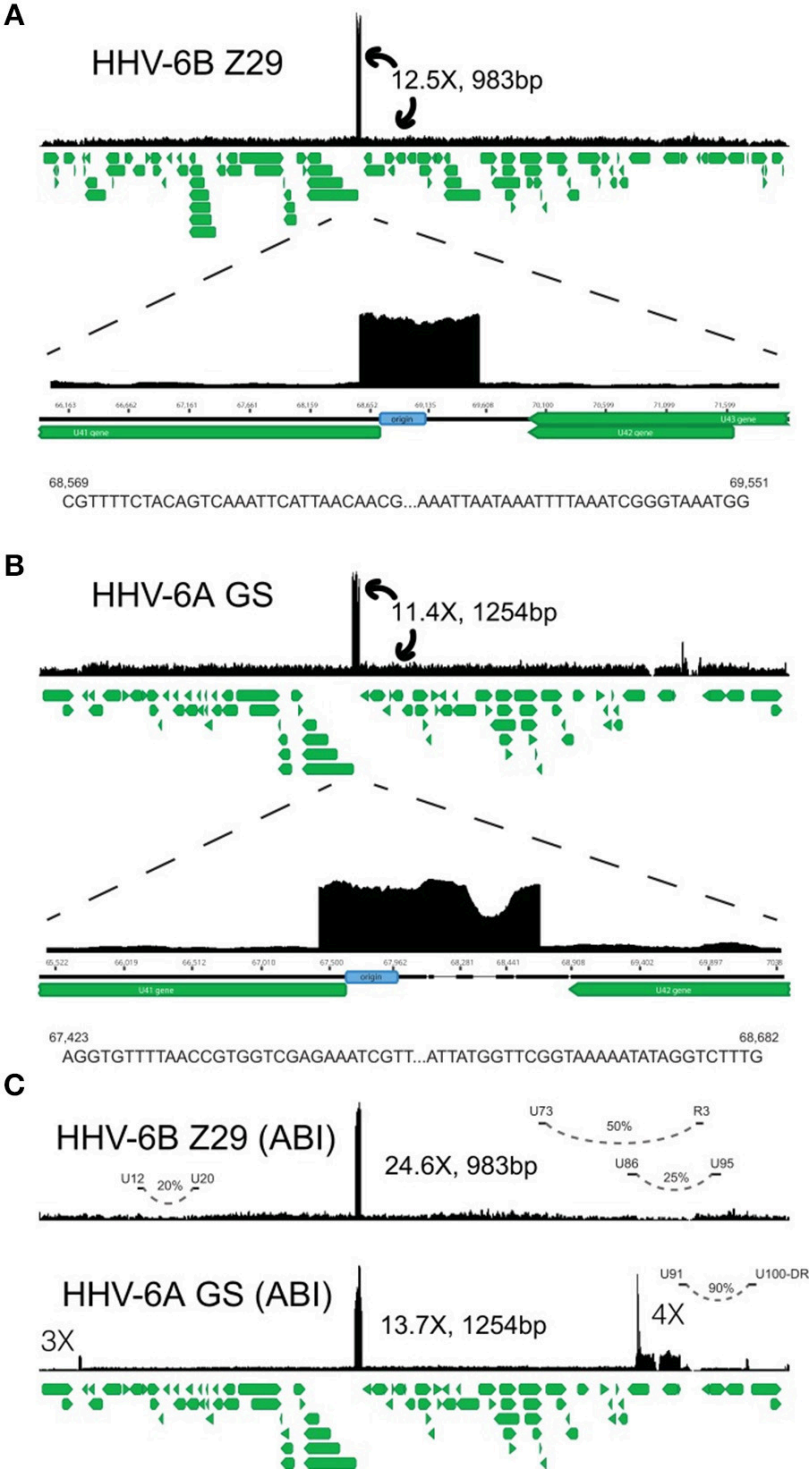
HHV-6A and HHV-6B genomic map. Representative coverage maps of HHV-6B Z29 and HHV-6A GS reference strains. Shotgun DNAsequencing reads from cultured virus were mapped to the NCBI HHV-6B and HHV-6A reference genomes, NC_000898 and NC_001664, respectively. The green stacked lines indicate the gene models for the respective viral species. **(A)** HHV-6B strain Z29 yielded a homogeneous 983-bp tandem repeat that was present at ~12.5 times higher coverage than the rest of the genome. Sequences at the 5 = and 3 = ends of the tandem repeat in strain Z29 are depicted and are different than those indicated previously (31). **(B)** HHV-6A strain GS yielded a heterogeneous 1,254-bp tandem repeat that was present at ~11.4 times higher coverage than the rest of the genome. Sequences at the 5 = and 3 = ends of the heterogeneous tandem repeat in strain GS are depicted. **(C)** ABI quantitative DNA material for HHV-6A GS and HHV-6B Z29 also demonstrated similar origin tandem repeats with additional loci with copy number differences in the GS strain. Long-distance rearrangements between U12 to U20, U73 to R3, U86 to U95, and the U91-to-U100/DR intergenic region are represented by curved dashed lines, and the estimated viral subpopulation containing the respective deletion is indicated by the percentage. From Greninger et al. (111).

Transfection of normal mouse and human cells with HHV-6 DNA fragments, and with the entire genome, led to the induction of tumors in nude mice, as initially shown by Puri, Razzaque et al. (4, 7, 161). Although the mechanism of transformation was not determined, loss of normal chromosomes and the presence of extra marker chromosomes were detected. In examining viral genes (Figure 2), Kashanchi et al. demonstrated that HHV-6 ORF-1 (DR-7) gene expression led to tumor production (113). The ORF-1-associated oncoprotein has been detected in non-Hodgkin lymphoma, glioblastoma, and in RS cells of Hodgkin lymphoma (9, 25, 99), and it is thought that it may act by binding to and inhibiting expression of the tumor suppressor p53, which can also be bound by the HHV-6 U14 protein (162). Specifically, HHV-6 infection is thought to inhibit p53 nuclear localization, thereby limiting its ability to inhibit cell growth and promote apoptosis (163). These findings support the possibility that HHV-6 is oncogenic (113).

Another HHV-6 protein, U24, has weaker associations with oncogenicity, but recent developments merit its mention. HNedd4L-WW3^*^ domain (human neural precursor cell expressed developmentally down-regulated protein 4-like) has newly been identified as a cognate ligand of the HHV-6A U24 protein, which is expressed in the early stages of infection (164). Notably, Nedd4 dysregulation has been observed in glioma (165, 166) and glioblastoma multiforme (167).

The HHV-6B immediate-early gene U95 protein interacts with GRIM-19 and is associated with loss of mitochondrial membrane potential (168). GRIM-19 is involved in the oxidative phosphorylation system and in regulation of cell death, and it is also bound by KSHV, HPV type 16, and simian virus 40 LT proteins, as well as CMV RNA (169). The HHV-6B U95 promoter is regulated by R3, a repetitive region of HHV-6B with NF-κB-binding sites (170). NF-κB is important in the control of cellular proliferation and survival. As noted previously, initial data pointed to possible HHV-6B-induced TLR3, Bcl-2, and NF-κB up-regulation as well as down-regulation of cleaved caspase 3 in rat pituitary adenoma cells. However, in order to distinguish causation from correlation, this requires follow up and use of HHV-6B rather than a viral mimic (57).

IHC performed on some cancer samples has revealed an absence of HHV-6 antigens in neoplastic cells, suggesting that if HHV-6 plays a role in neoplasia, it may participate indirectly (26), perhaps by modulating the tumor microenvironment or enhancing the potential for a primary tumorigenic virus to induce neoplasia (99). In one study, adult T cell leukemia cells that were persistently infected with HHV-6B triggered the growth of uninfected, neighboring cells, indicating that HHV-6 may affect inter-cellular signaling pathways to stimulate the growth of transformed cells that are not themselves infected (171). Both species have robust immunomodulatory abilities and are able to impact cytokine and chemokine expression and networks (172). The ways by which each virus may produce both immunosuppressive and inflammatory responses, both of which can impact carcinogenesis, are myriad and too extensive to comprehensively describe in this review.

Of interest, the HHV-6A/B gene U94 exhibits anti-tumorigenic effects in some *in-vitro* carcinoma models. Expression of HHV-6B U94 in breast cancer and cervical cancer cell lines decreased the migration and invasiveness of the cells *in vitro*, and the injection of U94-expressing cancer cells into mice resulted in reduced tumor growth and more limited vasculature in the resulting tumors (173). Earlier, HHV-6A was found to reduce lymphangiogenesis and angiogenesis *in vitro* (174) and limit migration of endothelial cells (174) and oligodendrocyte progenitor cells (175). As noted previously, U54 also inhibited breast cancer cell proliferation *in vitro* (142). While the express use of the U94 gene in vector form, in the absence of the rest of the genome, may hold potential in arresting the development of certain cancers, it is unclear what these anti-oncogenic properties mean when viewed holistically, and how this particular viral gene interacts with others that are expressed during the infectious course.

In spite of high molecular homology between the two HHV-6 species, it is obvious that their pathomechanisms, including carcinogenic and co-carcinogenic effects, are different. The two species utilize different receptors for cellular entry; consequently, their intracellular signal transduction pathways differ on a basic level. HHV-6A binds to CD46, which is a complement regulatory protein (176). CD46 signal transduction exerts positive regulation on T cell proliferation, regulatory T cell differentiation, and IL-10 production, which is regarded as immunosuppressive. Expression of CD46 is upregulated in many tumors (177).

HHV-6B, on the other hand, typically binds to CD134, also known as OX40 or tumor necrosis factor receptor superfamily member 4 (TNFRSF4) (178, 179). Binding of a ligand to CD134 blocks apoptosis, preventing the death of proliferating T cells and positively regulating B cell proliferation by modulating Th1 and Th2 mediated function (180, 181). These effects on the immune system might relate to immune dysregulation and lymphomagenesis.

Lately, there has been increased recognition of the importance of regulation of gene expression at the posttranscriptional level. Among small non-coding RNA (sncRNA) products, microRNA (miRNA) produced by both infected cells and by viruses are able to mutually regulate cellular and viral mRNA translation through base-pair complementarity. Abnormal activity of cellular miRNA has been associated with cancer and viral immune evasion. Viruses express functional miRNA that target both viral and cellular transcripts to suppress or destabilize mRNA. The net result of either process is reduced synthesis of proteins encoded by target mRNA.

Roseoloviruses, which are phylogenetically ancient viruses, have not developed miRNA-associated regulatory mechanisms as sophisticated as those of the gamma herpesviruses. Both EBV and KSHV encode viral miRNAs (44 and 25, respectively) that have been directly linked with malignant transformation. Of interest, some herpesvirus miRNA (EBV-miR-BART2-5p and KSHV-miR-K12-7), despite having evolved independently, suppress identical cellular mRNA targets, thus developing similar strategies to modulate host cell transformation (182).

Little attention has been paid to roseolovirus miRNA. Only recently, five novel HHV-6A encoded sncRNAs have been identified in Jjhan cells infected by strain U1102, one of which is a miRNA. This HHV-6 miRNA targets the HHV-6A immediate early (IE) gene U86 and is consequently named miR-U86. Overexpression of miR-U86 in transduced Jjhan cells results in significant growth retardation and reduced cell viability (183). Current data suggest that miR-U86 has no role in tumorigenesis. HHV-6A alters cellular miRNA expression in infected T cells, but too little information is currently available to draw conclusions about the relationship between these changes and oncogenesis (184).

Infection of Sup-T1 cells with HHV-6B, on the other hand, results in an abundant production of sncRNAs derived from either the direct repeat regions or the lytic origin of replication that give rise to smaller RNA species. Four miRNAs were identified and named hhv6b-miR-Ro6-1,−2,−3, and−6. Ro6-2 is a seed ortholog of the human miR-582-5p, which is upregulated in certain pituitary adenomas and downregulates expression of TGF-β (185). Similar to the HHV-8 miR-155 ortholog miR-K12, HHV-6B Ro6-2 may share a broad range of cellular mRNA targets with human miR-582-5p and may modulate the host-pathogen relationship. In cells (e.g., CD4^+^ T cells) that do not normally express cellular miRNA-582-5p, HHV-6B infection may lead to Ro6-2 miRNA-mediated suppression of TGF-β. HHV-6B encoded miRNAs are expressed in low abundance, similar to the oncogenic Marek's disease virus, but in contrast to other herpesviruses (186). All in all, the similarities between HHV-6B encoded miRNAs and oncogenic KSHV and human miRNAs suggest that they may play roles in tumorigenesis, though more work is needed to clarify their effects.

Roseoloviruses can also regulate cell-virus interactions through the release of microvesicles. Viruses have evolved to insert viral components into uninfected cells via microvesicles (MV) that are shed from the plasma membrane, as well as exosomes, which originate within microvesicular bodies (MVBs) and are released into the extracellular environment. Both can be isolated from any bodily fluid or excretion, and they can cross the blood-brain-barrier. They can include cellular and viral proteins, mRNAs, miRNAs, lipids, and carbohydrates. Once released, they can be taken up through phagocytosis or pinocytosis by neighboring or distant cells, where the contents can have direct consequences by manipulating gene expression of the recipient cells. HHV-6 infection dramatically increases MVB formation. HHV-6B redirects MHC class I molecules into MVBs, and along with gB viral proteins, they are released in exosomes. It is possible that the MHC molecules can assist viral entry in other cells (187). A reduction in MHC class I molecules may also contribute to ineffective immune targeting and clearance of transformed cells (188). In addition, HHV-6A infected HSB-2 cells tend to form MVBs, which can contain mature virions, and the small vesicles inside the MVBs carry the envelope glycoproteins gM and gB. It is presumed that viral glycoproteins expressed in exosomes may interact to form a virological synapse and promote the efficient spreading of HHV-6A from infected to uninfected cells. Furthermore, several reports on other viruses have shown T cell activation in response to exosomes secreted by antigen presenting cells (189).

Lately, studies on extracellular vesicles have indicated that they play a significant role in tumor progression (190). Although data on HHV-6-associated microvesicle pathomechanisms is limited, comparisons with other carcinogenic herpesviruses might help to elucidate their possible tumorigenic effects. EBV, for example, can introduce viral antigens, RNA, and growth factors into other cells. Unfortunately, the absence of active viral infection in these targeted cells may hamper the verification of a causal link between the virus and diseases in which viral infection has been implicated. KSHV was also found to modulate the microenvironment through packaging of viral factors in exosomes. The viral factors increased cell migration and IL-6 production, and promoted a switch to glycolytic metabolism in recipient endothelial cells (191), all of which have been implicated in carcinogenesis (192, 193). Further studies on HHV-6A and HHV-6B associated MVs and exosomes are required to verify their direct or indirect carcinogenic effects.

**Figure 3:**
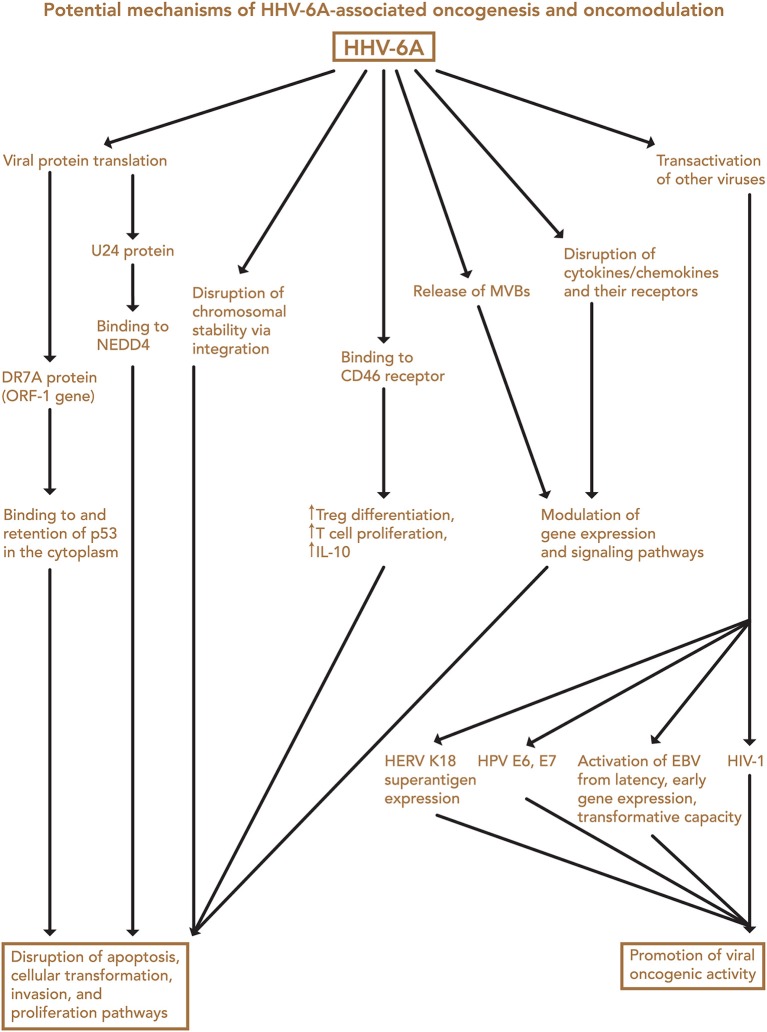
Potential mechanisms of HHV-6A-associated oncogenesis and oncomodulation. Several HHV-6A-mediated changes may contribute to disruption of apoptosis, cellular transformation, invasion, and proliferation pathways, and transactivation of other viruses may contribute to their oncogenic activity. EBV, Epstein-Barr virus; HERV, human endogenous retrovirus; HPV, human papillomavirus; HIV-1, human immunodeficiency virus-1; MVB, microvesicular bodies; Treg, regulatory T cell.

**Figure 4:**
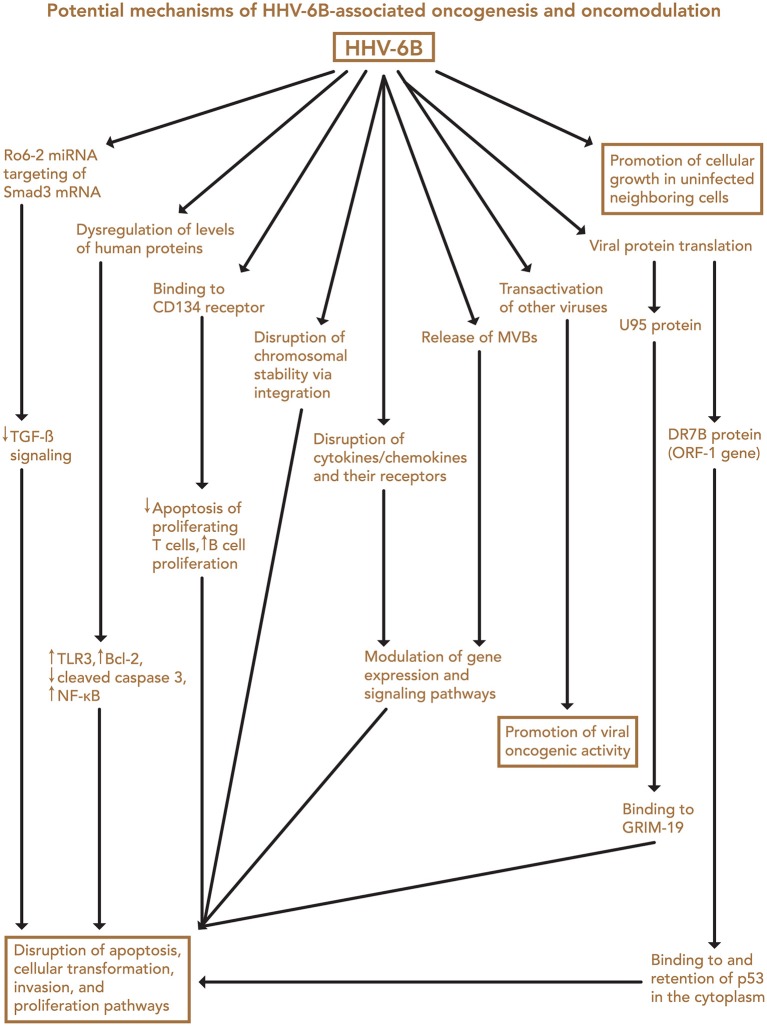
Potential mechanisms of HHV-6B-associated oncogenesis and oncomodulation. Several HHV-6B-mediated changes may contribute to disruption of apoptosis, cellular transformation, invasion, and proliferation pathways, and transactivation of other viruses may contribute to their oncogenic activity. In addition, HHV-6B-infected cells may promote cellular growth in neighboring uninfected cells through a presently unknown mechanism. MVB, microvesicular bodies.

Finally, both iciHHV-6 and community strains (71) of HHV-6 may affect chromosomal stability or transcription of genes associated with malignancy through integration into the cellular genome. Depending on the site of integration, telomeric disruption, loss of chromosomal integrity, and/or disruption of genes associated with cancers may result, in turn promoting oncogenesis. Proposed mechanisms of HHV-6-associated oncogenesis and oncomodulation are summarized in Figures 3, 4.

## Risk factors and molecular epidemiology

HHV-6 tends to reactivate during periods of stress and immunosuppression. Like HHV-8, immunosuppressive conditions- present through inherited, environmental, or age-associated means may be key to pathogenic reactivation of HHV-6 resulting in oncogenic changes. While immunosuppression triggers viral reactivation from latency, both HHV-6A and HHV-6B are also immunomodulatory viruses that affect the functions of natural killer cells and T cells, as well as other immune cells, cytokines, and chemokines (172). In establishing persistence, the viruses employ strategies to evade detection and elimination by immune cells, which can result in down-modulation of immune cell activities. The effects of infection are myriad, time-dependent, and vary across individuals. Genetic factors are likely to affect the extent of HHV-6-asscoiated immunomodulation and outcomes during and after primary HHV-6 infection, although the contribution of genetics in HHV-6-associated illness is largely unknown. Likewise, environmental influences may impact the types of changes that the viral infection induces in individuals. In terms of HHV-6A and HHV-6B themselves, it is possible that certain variants are more prone to contributing to oncogenesis or in dampening the immune response.

HHV-6B reactivates frequently in patients after receiving hematopoietic stem cell transplantation for hematological malignancies (194) and has been shown to impair late reconstitution of CD4^+^ T cells, likely by affecting thymopoiesis (195–197). A weaker immune response as a consequence of HHV-6 infection may allow for circumstances beneficial for the continuation of neoplasia, as immune cells may be less effective in detecting and destroying abnormal cells. It has been suggested that HHV-6 infection contributes to the progression of GIC through the promotion of lymphopenia and immunosuppression (134), and in some instances, atypical lymphoproliferative disorders progress to overt malignant lymphoma, particularly in the setting of immunodeficiency (198, 199). Virus-mediated dysregulation of cell proliferation, differentiation, and cell death may play a role (200, 201).

While both viruses have shown oncogenic potential themselves, HHV-6A and –B-mediated transactivation of other oncogenic agents may also play an indirect role in tumorigenesis. In general, herpesviruses, but especially beta herpesviruses, are known for transactivating each other and several other heterologous viruses. HHV-6A U16 and U30 gene products transactivate E6 and E7 of HPV-16 in cervical epithelial cells (149). Additionally, HHV-6A activates EBV from latency (202), increases EBV early gene expression (203), and bolsters its transformative capacity. Meanwhile, EBV renders B cells susceptible to HHV-6 infection (203, 204). HHV-6A has also been shown to transactivate the long terminal repeat of HIV-1 in double infected cells (205), and several gene products of HHV-6A have been identified as possessing HIV-1 activating potential independently of each other (206). Soluble mediators (e.g., TNF-α) released from HHV-6A–infected lymphoid cells also upregulated HIV-1 infection in other cells carrying the virus (207). All of these effects could consequently facilitate AIDS and AIDS–associated tumor progression. HHV-6A (208) and HHV-6B (209) activate human endogenous retrovirus (HERV) K18 superantigen expression, and some HERV species have been implicated in teratocarcinoma and other germline tumors.

Exploring biomarkers and performing functional studies to investigate interactions between HHV-6 activity and genetic variants related to molecular/cellular pathways involved in immune surveillance and clearance of transformed cells, as well as to susceptibility to other infections, will be valuable going forward.

## Concluding remarks

Detection of viral RNA/DNA in tumor tissue by PCR and sequencing is the first step in elucidating the role of a virus in tumors. Given the presence of numerous reactive inflammatory cells and stromal cells within tumor tissue, PCR positivity may arise from non-tumor cells. As such, the next step would be direct localization of virus to tumor cells by immunohistochemistry, *in-situ* hybridization, and/or electron microscopy. These techniques require high quality tumor tissue, high sensitivity and specificity of the detection reagents, and simultaneous running of proper positive and negative controls. Even if the virus is localized to tumor cells, the percent positivity of tumor cells is likely important since one would expect that if the virus is involved in tumor growth, then it should be present in at least a significant fraction of tumor cells.

Cancer development follows a multistep selection course with acquisition of genetic defects leading from a benign polyclonal process to a malignant monoclonal process (210). In some settings, HHV-6A/B may play a supportive role in this process. Even if not overtly oncogenic in terms of inducing or activating oncogenes in susceptible cells, HHV-6 may indirectly stimulate growth and/or block apoptosis in infected cells, interfere with epigenetic regulation and post-translational events, or potentiate the role of other viruses, in susceptible individuals (211). Although no definitive evidence of a direct role for HHV-6 as a tumorigenic virus has been produced, further investigation is warranted, especially for nodular sclerosis Hodgkin Lymphoma, glial tumors, gastrointestinal tumors, and oral cancers.

## Author contributions

EE, EL, JP, JO, GK, JC, TP, DA, and SH: all contributed to the content of the manuscript and edited as needed.

### Conflict of interest statement

The authors declare that the research was conducted in the absence of any commercial or financial relationships that could be construed as a potential conflict of interest.
